# Translation of the Debriefing Assessment for Simulation in Healthcare in Portuguese and cross-cultural adaptation for Portugal and Brazil

**DOI:** 10.1186/s41077-021-00175-z

**Published:** 2021-07-07

**Authors:** Thomaz Bittencourt Couto, Francisco Maio Matos, Paula Dias de Toledo Rodovalho, Mary Fey, Robert Simon, Sacha Muller-Botti

**Affiliations:** 1 Centro de Simulação Realística, Hospital Israelita Albert Einstein, Av. Albert Einstein, 627/701, Bloco A–1o subsolo, Sao Paulo, SP CEP 05651-901 Brazil; 2 Centro de Simulação Biomédica, Centro Hospitalar e Universitário de Coimbra, Coimbra, Portugal; 3 Center for Medical Simulation, Boston, USA; 4 Hunter New England Simulation Centre, John Hunter Hospital, New Lambton Heights, New South Wales Australia

**Keywords:** Translation, Cross-cultural adaptation, Assessment, Debriefing

## Abstract

**Background:**

Debriefing Assessment for Simulation in Healthcare (DASH©) is an instrument to assist in developing and evaluating debriefing skills. The objectives of this study were to translate the DASH from English to Portuguese and to conduct a cross-cultural adaptation of this translated instrument for Portugal and Brazil.

**Methods:**

A forward translation of the DASH score sheets and Rater’s Handbook was accomplished and reviewed by authors from both Portuguese-speaking countries to reach the consensus harmonized version. A backward translation was reviewed by the original authors and discussed with the authors to produce the approved harmonized translation. This was then tested through a questionnaire to assess clarity, comprehensiveness, appropriateness, and cultural relevance among 10 simulation specialists from Portugal and Brazil.

**Results:**

During the forward translation, 19 discrepancies were detected in the Portuguese DASH. After backward translation, 7 discrepancies were discussed and harmonized. All 10 simulation specialists from both countries reviewed the harmonized translation and made 70 suggestions, 64 of which were incorporated in the instrument after discussion among authors.

**Conclusions:**

The translated DASH has undergone translation to Portuguese and a cross-cultural adaptation across Portugal and Brazil. It may be used to assess debriefings in healthcare settings in these countries.

**Supplementary Information:**

The online version contains supplementary material available at 10.1186/s41077-021-00175-z.

## Background

Debriefing is a core element of simulation-based learning [1]. After participating in a simulated scenario, debriefing allows participants to reflect about the case, which is a crucial step in the experiential learning process [2]. The debriefing provides a forum for learners to identify performance gaps, discuss areas for improvement, and consolidate lessons that can be applied in later practice [3]. Given the pivotal role of debriefing, a tool that yields data that support valid judgments of an instructor’s debriefing competence has the potential to greatly facilitate faculty training [4].

There are only two debriefing assessment tools currently available in Portuguese. One instrument is the Debriefing Experience Scale, developed in the USA by Reed [5], created to measure the experience of nursing students in debriefing. It was translated and adapted to Portuguese by Almeida in 2016 [6]. The other instrument is *Escala de Avaliação do Debriefing associado à Simulação* (Debriefing Assessment Scale Associated to Simulation), created by Coutinho to assess debriefing using Portuguese simulation specialists and nursing studies [7]. Both scales assess debriefing quality from the student’s perspective, so there is a demand for instruments in Portuguese assessing debriefing from the point of view of the instructor. Objective Structured Assessment of Debriefing (OSAD) was described originally in surgery literature [8], with modifications to be used in pediatrics [9] and online [10]. It includes a list of eight features essential to an effective debriefing, scored in a 5-point Likert scale. Peer-Assessment Debriefing Instrument (PADI) was developed with a component of self-evaluation and facilitator assessment, assessing eight aspects of planning and conducting a simulation debriefing in a 4-point Likert scale [11].

The Debriefing Assessment for Simulation in Healthcare (DASH) tool was designed to assist in evaluating faculty skills in healthcare simulation debriefings. It is a behaviorally anchored rating scale that, when used by trained raters, has shown interrater reliability and internal consistency, with an overall intraclass correlation coefficient for the combined elements of 0.74 and a Cronbach α of 0.89 across the 114 webinar participants rating three standardized debriefing sessions [4]. There are three versions of the DASH: DASH Rater Version—designed for trained raters to rate instructors, DASH Student Version—designed for students to rate their instructors, and DASH Instructor Version—designed for instructors to rate themselves. Besides these tools, the DASH Rater’s Handbook provides instructions and examples of instructors’ behaviors to assist in rating [4]. DASH has some advantages compared to other scales available to assess the facilitator during debriefing, because it was created to assess debriefing from different perspectives and applies to simulations in a variety of domains and disciplines [4].

With the international expansion of simulation-based training, there is a need to translate and validate tools like DASH in other languages, such as Portuguese [12]. Portuguese is the ninth most spoken language in the world, with roughly 250 million speakers [9, 10]. The instrument’s translation and back translation are the first steps to use an instrument in another language.

Portuguese spoken in Portugal (European Portuguese) and Brazil (Brazilian Portuguese) have significant lexical and cultural differences [13, 14]. When translating an instrument using the same language in different countries, there is a choice between a country-specific or a universal approach. Although more labor-intensive, the universal approach has the advantage of reducing differences that could impact the understanding of the document [15]. Given the cultural differences between the Portuguese language spoken in Brazil and Portugal, a cross-cultural adaptation was essential to ensure that a worldwide Portuguese translation was available.

Our study was represented only by two countries, Brazil and Portugal. These are responsible for most of the scientific production from Portuguese-speaking countries, being the only two Portuguese-speaking countries among top 50 countries in scientific production according to the Scimago Journal and Country Rank [16]. They are also the two countries with structured healthcare simulation societies, ABRASSIM, and SPSIM.

The objective of this study was to translate the original three versions of DASH© and the Rater’s Handbook from English to Portuguese and conduct a cross-cultural adaptation across Brazil and Portugal, aiming to produce a Portuguese version of the DASH tool that can capture the intended meaning of the English version, and it is well understood by Portuguese-speaking users in Brazil and Portugal.

## Methods

**Fig. 1:**
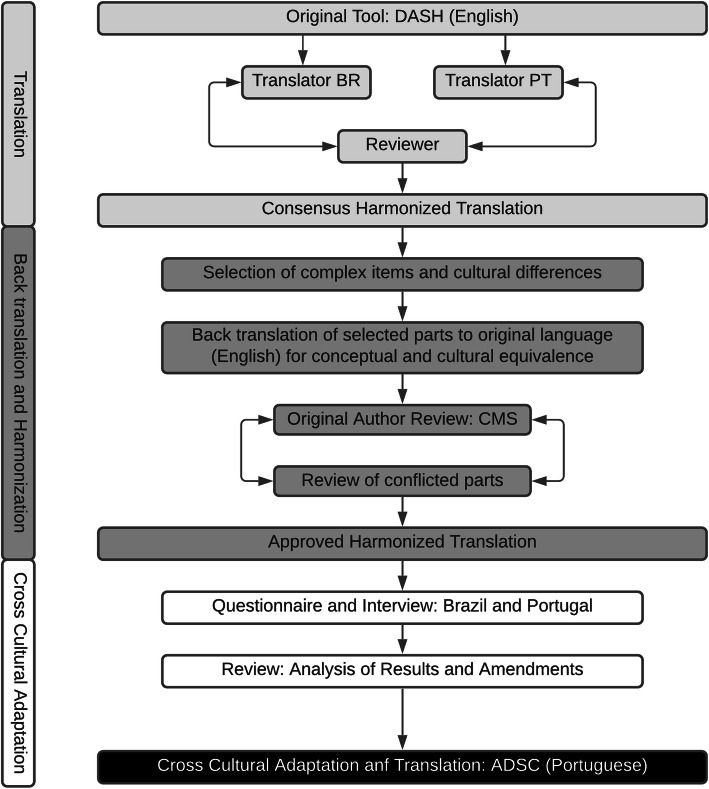
Schematic overview of the translation and cross-cultural review process, based on methodology from Spanish DASH translation [12]

An overview of the translation and the cross-cultural review process is shown in Fig. 1. This was based on the diverse available guidelines for translation [17–19] and cultural adaptation, and on the recently published Spanish translation of the DASH tool [12].

### Original document

There are three versions of the DASH rating instrument: rater version (DASH-RV), student version (DASH-SV), and instructor version (DASH-IV), and a Rater's Handbook, all of which were translated in this study. Each score sheet is composed of 6 elements, scored via a 7-point rating scale, describing distinct debriefer behaviors. After describing and defining an element, behaviorally anchored dimensions are included to further enhance the rater’s understanding of each element. There are 23 dimensions distributed across the 6 elements. Two different score sheets are available for each version; a short form which scores elements and a long form which scores elements and dimensions [4, 12].

The DASH Rater’s Handbook provides instructions and examples reflecting debriefer’s behavior to assist with the rating of different elements and dimensions. Within each of the 23 dimensions, observable positive and negative behavior examples are provided. Table 1 provides the structure of the DASH elements and dimensions [4].

**Table 1:** Elements and dimensions of the DASH

DASH element	Element dimensions
1. Establishes an engaging learning environment	• Clarifies course objectives, environment, confidentiality, roles, and expectations • Establishes a “fiction contract” with participants • Attends to logistic details • Conveys a commitment to respecting learners and understanding their perspective
2. Maintains an engaging learning environment	• Clarifies debriefing objectives, roles, and expectations • Helps participants engage in a limited-realism context • Conveys respect for learners and concern for their psychologic safety
3. Structures the debriefing in an organized way	• Encourages trainees to express their reactions and, if needed, orients them to what happened in the simulation, near the beginning • Guides analysis of the trainees’ performance during the middle of the session • Collaborates with participants to summarize learning from the session near the end
4. Provokes engaging discussions and uses concrete examples and outcomes as the basis for inquiry and discussion	• Reveals own reasoning and judgments • Facilitates discussion through verbal and nonverbal techniques • Uses video, replay, and review devices (if available) • Recognizes and manages the upset participant
5. Identifies and explores performance gaps	• Provides feedback on performance • Explores the source of the performance gap
6. Helps trainees achieve or sustain good future performance	• Helps close the performance gap through discussion and teaching • Demonstrates firm grasp of the subject • Meets the important objectives of the session

### Forward translation

As suggested by Sousa and Rojjanasrirat [20], two translators independently translated all the components of the DASH tool. The translators were fluent in the language of the original document (English) and needed to be native speakers of the target language (Portuguese). Both translators had some experience with simulation in healthcare concepts. One translator (PR) is a Brazilian nurse educator working full time with simulation for the last 7 years, with experience in teaching simulation courses for graduate, post-graduate students, and faculty. The other translator (FM) is a medical doctor from Portugal, coordinates a simulation center, and has more than a decade of simulation experience, with experience in teaching simulation courses across Europe.

The reviewer is a fluent English speaker whose native language is Portuguese (TC). He is the medical educator of a large simulation program accredited by the Society for Simulation in Healthcare (SSH) in Brazil, is a Certified Healthcare Simulation Educator-Advanced by SSH, conducts research, and teaches simulation-based courses regularly in Portuguese and English.

### Consensus harmonized translation

Between the translators and the reviewer, the 6 DASH elements underwent 5 iterative revisions until all parties agreed that the translation was accurate. Consistent with the World Health Organization Translation and Adaptation of Instruments [19], the aim was the conceptual equivalent of a word or phrase, not a literal translation. Translators strived to be simple, clear, and concise, avoiding long sentences with many clauses. Translators and reviewer met multiple times to achieve a common language in the rater form and to replicate this on the other score sheets and the manual. Additional file 5 shows different versions of the rater form and back translation. Once an agreement was achieved, the document was considered the “Consensus Harmonized Translation”. The new names of the DASH became Portuguese DASH (translated as *Avaliação do Debriefing para Simulação Clínica*–Evaluation of Debriefing for Clinical Simulation).

### Back translation

All score sheets, including the 6 DASH elements, were back translated from Portuguese to English. In a design similar to the Spanish DASH [12], only the Elements 2 and 5 of the Raters’ Handbook were back translated. These 2 elements were considered the most essential and relevant by the original authors. If only minor changes were needed, it was thought that it was appropriate to extrapolate the translation to the rest of the document. However, if the 2 elements needed extensive rework, all 6 elements would need to be back translated and reviewed.

The back translation was undertaken by a professional bilingual translator. He is a science translator (English/Portuguese and Portuguese/English), writer, and editor with experience in translating scientific, medical, and technical materials for publications from different fields such as nursing, plastic surgery, general medicine, orthopedics, and psychology.

### Approved harmonized translation

The authors of the original document in English performed an expert review of the back translation. For any discrepancies detected, the forward translators, reviewer, and back translator had discussions to determine the source of the problem. A table was created to outline the conflicts detected between the back translation and the original document. Each conflict was categorized as a term, concept, or syntax error. The source of the conflict was identified to determine whether the forward translation needed to be modified. Amendments were made until a satisfactory version was reached. This created the approved harmonized translation [21].

### Cross-cultural adaptation

The approved harmonized translation (Portuguese DASH) was evaluated by simulation specialists to assess clarity, comprehensiveness, appropriateness, and cultural relevance among 10 subjects from both countries, 5 from Brazil, and 5 from Portugal. A standard sample size calculation is difficult to apply because of the nature of the analysis. When focusing on qualitative data, it has been shown that 5 users revealed an average of 85% of issues on usability testing procedures [21]. Simulation specialists who were invited to collaborate evaluating the harmonized translation were required to have a master’s level degree, at least 7 years of professional experience with healthcare simulation, and were identified through contacts with the Brazilian (ABRASSIM) and Portuguese (SPSIM) simulation societies.

The specialists were asked to evaluate the harmonized document, analyzing their understanding of terms, key concepts, verb tense, and general writing. Space was allocated for the participants to write any concepts that were not clear or that they thought were inappropriate for each of the forms and each element of the Raters’ manual.

### Analysis of results and amendments

All responses were reviewed, and the following criteria were used to decide which suggested changes would be applied to the forward translation:

- Repeated suggestion from participants from different countries.
- The suggestion significantly improved the understanding of the translated document in all countries.
- It was a true representation of the original English document (DASH).
- It conveyed the same meanings and concepts as the DASH.

## Results

### Forward translation and harmonization

Forward translation results are displayed as Additional file 5, with Brazilian and European Portuguese versions of translation and harmonized version of the three scoring sheets. Between the translators and the reviewer, the DASH scoring sheets, and Handbook underwent five iterative revisions until all parties agreed that the translation was accurate. Translators and reviewer met multiple times online and twice in person to achieve a common language and to replicate this on all score sheets and the manual.

### Back translation

Once the harmonized version of the Handbook manual was reached, it was back translated by the professional scientific translator (Additional file 6). Elements 2 and 5 from the back translation were compared with the original English document by the original DASH authors. A total of 19 discrepancies were found. Detailed conflicts between back translation and original DASH are shown in the supplemental table (Additional file 7). The distribution and number of changes incorporated into all versions, handbook, and score sheets are shown in Table 2.

**Table 2:** Discrepancies detected between DASH and back translation of elements 2 and 5

Portuguese	
• 19 discrepancies	
○ Term: 14 (73.7%)	
○ Syntax: 2 (10.5%)	
○ Concept: 3 (15.8%)	
• 12 (63.2%) discrepancies were originated in the back translation and, therefore, did not incur any necessary changes to the consensus harmonized translation	
• 7 (31.6%) discrepancies were originated in the forward translation and prompted necessary changes	
○ Term: 4 (57.1%)	
○ Syntax: 2 (28.6%)	
○ Concept: 1 (14.3%)	

### Cross-cultural adaptation

Five participants for each country were sent the questionnaire and all (100%) responded after reviewing the score sheets and Handbook.

There were 70 recommendations from the open-ended questions; 13 were related to the score sheets and 57 to the Handbook. Of these recommendations, 41 (58.6%) were terms, 2 were (2.9%) concept, and 27 (38.6%) were syntax.

Using the criteria described in the methodology, 64 (91.4%) of the recommended changes were applied to the approved harmonized translation.

There were two terms that were not possible to reconcile initially. The orthography for teamwork in “trabalho em equipe” in Brazil and “trabalho em equipa” in Portugal and the word perspective, which is spelled “perspectiva” in Brazil and “perspetiva” in Portugal. After discussion with the original authors´ of the DASH, since the difference in spelling did not impact the users´ ability to understand the meaning of these terms, a compromise was reached, maintaining the terms “equipa” in the European spelling and “perspectiva” in the Brazilian spelling (Additional files 1, 2, 3, and 4).

## Discussion

The results support the equivalence between the Portuguese and English DASH. Thus, we propose the amended versions of the documents can be utilized for the transcultural assessment of debriefings in Brazil and Portugal.

Methods used in this study were adapted from the Spanish version of DASH [12] and the proposed guideline from Sousa and Rojjanasrirat [20]. The methods included multiple opportunities for translation, back translation, cultural understanding, and harmonization which are desired to produce, not only linguistic, but also cultural equivalence [22, 23].

The comparison between the back translation and the original document, done by the original authors of the DASH, identified several discrepancies that required minor changes in the Portuguese forward translation, mostly related to specific terms and syntax. The authors of this paper, together with the original authors of the DASH, considered that 19 discrepancies in total between the forward and back-translation were not significant to warrant the back-translation of the whole DASH Handbook. It was accepted that an extrapolation could be made and safely assume that the forward translation of the DASH Handbook was accurate enough to be utilized. This was consistent with the results presented by the Spanish DASH, which also had few modifications suggested, mostly related to terms and syntax [12].

The results of the evaluation by simulation specialists suggested more changes than the analysis of the back translation, highlighting the importance of cross-cultural adaptation. This was similar to the results from the Spanish DASH. However, the vast majority of changes suggested in the cross-cultural adaptation in the Portuguese version of DASH were accepted, in contrast to the Spanish DASH, which had only 27% of suggestions applied to the final harmonized version [12]. This likely occurred due to the more experienced nature of the reviewers of the Portuguese DASH, who made more assertive suggestions. This probably also explains the very low discrepancies in concept suggestions in the cross-cultural adaptation.

Limitations of this study include the fact of our forward translation was done by native Portuguese speakers, as we were unable to locate bilingual native speakers with simulation knowledge to contribute to the study. Back translation was also performed by only one person, who is not a native English speaker, even though this was performed by a professional bilingual translator with extensive experience in scientific translation. Additionally, the translated instrument has not had psychometric studies and, therefore, we cannot guarantee its scientific accuracy for interrater reliability.

Future research studies include gathering evidence from a larger and more varied sample of Portuguese-speaking debriefers. In a similar fashion to what was already done for the original DASH instrument [4], studies to develop the psychometric properties statistics about interrater reliability and internal consistency are needed. It is necessary to gather evidence of construct validity of the Portuguese version by evaluating its ability to detect variations in the quality of debriefings in different simulation settings.

## Conclusions

The translated DASH has undergone translation to Portuguese and a cross-cultural adaptation across Portugal and Brazil. It may be used to assess debriefings in healthcare settings in these countries.

The Portuguese and English version of the DASH Handbook and Scoring Sheets are available for download at https://harvardmedsim. org/debriefing-assessment-for-simulation-in-healthcare-dash/

The Portuguese scoring sheets, Portuguese Rater’s Handbook, different translations of scoring sheets, back translation of elements 2 and 5 and supplemental table with translation conflicts are available as additional files.

## Supplementary Information

**Additional file 1.** Portuguese Rater Version.

**Additional file 2.** Portuguese Student Version.

**Additional file 3.** Portuguese Instructor Version.

**Additional file 4.** Portuguese DASH Rater's Handbook.

**Additional file 5.** Different translations of DASH rater, student and instructor version.

**Additional file 6.** Back translation of elements 2 and 5.

**Additional file 7: Supplemental table.** Conflicts detected between DASH and Back Translation of Elements 2 and 5.

## Data Availability

All data generated or analyzed during this study are included in this published article.

## Abbreviations

DASH: Debriefing Assessment for Simulation in Healthcare
PT: Portuguese
RV: Rater’s version
SV: Student’s version
IV: Instructor’s version
ABRASSIM: Brazilian Healthcare Simulation Association
SPSIM: Portuguese Society for Simulation
