# Supplementary material for: Translation of the Debriefing Assessment for Simulation in Healthcare in Portuguese and cross-cultural adaptation for Portugal and Brazil
Source: Adv Simul (Lond). 2021 Jul 7;6:25. doi: 10.1186/s41077-021-00175-z (PMC8265112; doi:10.1186/s41077-021-00175-z)
Supplement: Supplementary file 1 — Additional file 1. Portuguese Rater Version. [file 41077_2021_175_MOESM1_ESM.pdf]

DASH - *Debriefing Assessment for Simulation in Healthcare*©
Avaliação do *Debriefing* em Simulação Clínica – Classificação

Instruções: Classifique a qualidade do *debriefing* utilizando escalas de eficácia com seis Elementos. O Elemento 1 classifica a introdução da atividade de simulação e não será classificado se não observou a introdução. Os Elementos abrangem Dimensões e Comportamentos pertinentes para o *debriefing* como definido no "Manual do Avaliador do DASH". Em cada Elemento, o *debriefing* pode variar de Excelente a Prejudicial. Repare que a classificação geral dos Elementos **não** é a média das classificações para cada Dimensão ou Comportamento. Pense de formal global e não matematicamente quando considera o impacto das Dimensões, que podem não ter o mesmo peso. O avaliador deve ponderar as Dimensões da forma mais adequada, baseado na **visão global do Elemento**. Se não for possível avaliar uma Dimensão (ex: como se lidou com um participante descontente, se ninguém ficou descontente), ignore e não permita que isso influencie a sua avaliação.

Escala de Pontuação

Classificação	**1**	**2**	**3**	**4**	**5**	**6**	**7**
Descrição	**Extremamente ineficaz**<br>**Extremamente insatisfatório**	Consistentemente ineficaz<br>Consistentemente insatisfatório	Ineficaz<br>Insatisfatório	Pouco eficaz<br>Satisfatório	Bastante eficaz<br>Bom	Consistentemente eficaz<br>Muito Bom	**Extremamente eficaz**<br>**Excelente**

O Elemento 1 avalia a introdução no início de um exercício de simulação.
(Este Elemento deve ser ignorado se o avaliador não observou a introdução do curso.)

Elemento 1 **Classificação Elemento 1**
Estabelece um ambiente envolvente de aprendizagem.
____

- Esclarece todas as questões relacionadas com os objetivos da atividade, ambiente, confidencialidade, papéis e expectativas.
- Estabelece um "contrato de ficção" com os participantes.
- Esclarece detalhes logísticos.
- Define o compromisso de respeitar os participantes e compreender as suas perspectivas.

Os Elementos 2 a 6 avaliam o debriefing.

Elemento 2 **Classificação Elemento 2**
Mantém um ambiente envolvente para a aprendizagem.
____

- Esclarece os objetivos, papéis e expectativas do *debriefing.*
- Ajuda os participantes a envolverem-se num contexto de realismo limitado.
- Mostra respeito pelos participantes e preocupa-se com a sua segurança psicológica.

Elemento 3 **Classificação Elemento 3**
Estrutura o debriefing de forma organizada.
____

- Encoraja os participantes a partilhar as suas reações e, se necessário, orienta a sucessão de eventos do exercício.
- Orienta a análise do desempenho dos participantes durante o *debriefing*.
- Colabora com os participantes no resumo da aprendizagem da sessão que termina.

Elemento 4 **Classificação Elemento 4**

Incentiva discussões profundas.

____

- Usa exemplos e resultados concretos como base para os seus comentários e discussão.
- Revela o seu raciocínio e opinião.
- Facilita a discussão com técnicas de linguagem verbal e não verbal.
- Usa vídeos ou dados gravados (se disponíveis).
- Reconhece e aborda adequadamente participantes insatisfeitos.

Elemento 5 **Classificação Elemento 5**

Identifica e explora falhas de desempenho.

____

- Comenta o desempenho.
- Explora a origem das falhas de desempenho.

Elemento 6 **Classificação Elemento 6**

Ajuda os participantes a perceber como podem melhorar ou manter o seu desempenho.

____

- Ajuda no esclarecimento das falhas de desempenho através de discussão e ensino.
- Demonstra domínio de conhecimento sobre o tema.
- Aborda todos os pontos importantes.

Direitos Autorais. *Center for Medical Simulation*, www.harvardmedsim.org, 2011.
