# Supplementary material for: Translation of the Debriefing Assessment for Simulation in Healthcare in Portuguese and cross-cultural adaptation for Portugal and Brazil
Source: Adv Simul (Lond). 2021 Jul 7;6:25. doi: 10.1186/s41077-021-00175-z (PMC8265112; doi:10.1186/s41077-021-00175-z)
Supplement: Supplementary file 2 — Additional file 2. Portuguese Student Version. [file 41077_2021_175_MOESM2_ESM.pdf]

DASH - *Debriefing Assessment for Simulation in Healthcare©*
Avaliação de Debriefing em Simulação Clínica – Versão do Participante

Instruções: Pede-se que resuma a sua avaliação sobre a introdução e *debriefing* desta atividade de simulação. Utilize a seguinte escala para classificar cada um dos seis "Elementos". Cada Elemento é composto por diferentes Comportamentos do facilitador, descritos abaixo. Se um dos Comportamentos listados for impossível de avaliar (ex: como o facilitador lidou com pessoas descontentes, se ninguém esteve descontente), deixe o espaço em branco e não permita que isso influencie a sua avaliação. O facilitador pode ter sido mais ou menos bem-sucedido dentro de cada Elemento. A classificação do Elemento representa a sua impressão **geral** do desempenho do facilitador no Elemento em questão. Faça o possível para classificar a **eficácia geral de todo o Elemento**, guiado pela observação dos comportamentos únicos que o definem.

Escala de Pontuação

Classificação	**1**	**2**	**3**	**4**	**5**	**6**	**7**
Descrição	**Extremamente ineficaz<br>Extremamente insatisfatório**	Consistentemente ineficaz<br>Consistentemente insatisfatório	Ineficaz<br>Insatisfatório	Pouco eficaz<br>Satisfatório	Bastante eficaz<br>Bom	Consistentemente eficaz<br>Muito Bom	**Extremamente eficaz<br>Excelente**

O Elemento 1 avalia a introdução no início do exercício de simulação.

Ignore este elemento se não tiver participado na introdução.

Se não existiu introdução e sentiu que deveria ter existido para orientá-lo, a sua avaliação deverá refletir isso.

Elemento 1 **Classificação Elemento 1**
O facilitador preparou o cenário para uma experiência envolvente de aprendizagem.
____

- O facilitador apresentou-se, descreveu o local do cenário, expôs o esperado durante a atividade e os objetivos de aprendizagem.
- O facilitador expôs os pontos fortes e as limitações da simulação e o que se pode fazer para aproveitar ao máximo a experiência em simulação.
- O facilitador informou os detalhes logísticos necessários, como localização dos recursos sanitários, disponibilidade de alimentação e programa.
- O facilitador estimulou-o a partilhar os seus pensamentos e questões sobre o caso simulado e o *debriefing* e reforçou que não iriam existir constrangimentos durante o processo.

Os Elementos 2 a 6 avaliam o *debriefing*.

Elemento 2 **Classificação Elemento 2**
O facilitador manteve um ambiente envolvente para a aprendizagem.
____

- O facilitador esclareceu os objetivos do *debriefing*, o que era esperado dos participantes e qual o seu papel no *debriefing*.
- O facilitador reconheceu os problemas relacionados com o realismo e ajudou-o a aprender apesar de se tratarem de casos simulados.
- Sentiu que o facilitador respeitou os participantes.
- Os participantes puderam partilhar pensamentos e emoções sem medo de se sentirem constrangidos ou inferiorizados.

Elemento 3 **Classificação Elemento 3**
O facilitador estruturou o debriefing de forma organizada. __________

- A conversa progrediu de forma lógica em vez de saltar de ponto em ponto.
- No início do *debriefing,* foi encorajado a partilhar as suas reações genuínas relativamente ao caso e o facilitador considerou-as de forma séria.
- Durante o *debriefing*, o facilitador ajudou-o a analisar as ações e pensamentos enquanto revia o caso.
- No final, foi feito um resumo do caso no qual o facilitador o ajudou a integrar todas as observações e **enquadrou o caso de diferentes maneiras** para que possa melhorar a sua prática clínica futura.

Elemento 4 **Classificação Elemento 4**
O facilitador provocou discussões profundas que o levaram a refletir sobre o seu desempenho. __________

- O facilitador usou exemplos concretos – não apenas comentários abstratos e generalizados – para fazê-lo refletir sobre o seu desempenho.
- O ponto de vista do facilitador foi claro; não teve que adivinhar o seu pensamento.
- O facilitador ouviu e fez com que os participantes se sentissem ouvidos ao tentar incluir todos na discussão, interpretar os comentários e utilizar linguagem não verbal, como olhar nos olhos dos participantes ou acenar com a cabeça.
- O facilitador usou vídeos ou dados gravados para apoiar a análise e aprendizagem.
- Se alguém ficou descontente no *debriefing*, o facilitador foi respeitador e construtivo ao tentar ajudá-lo a lidar com a situação.

Elemento 5 **Classificação Elemento 5**
O facilitador identificou o que fizemos melhor e pior – e porquê. __________

- Recebeu comentários concretos sobre o seu desempenho e/ou do grupo baseados em declarações objetivas e honestas do facilitador.
- O facilitador ajudou a explorar o que pensava ou o que tentava alcançar nos momentos chave.

Elemento 6 **Classificação Elemento 6**
Ajudou os participantes a perceber como podem melhorar ou manter um bom desempenho. __________

- O facilitador ajudou-o a aprender como pode melhorar deficiências e/ou como repetir um bom desempenho.
- O facilitador era experiente e usou o seu conhecimento para o ajudar a perceber como pode melhorar o seu desempenho no futuro.
- O facilitador garantiu que todos os pontos importantes foram abordados.

Direitos Autorais. *Center for Medical Simulation*, www.harvardmedsim.org, 2011.
