# Supplementary material for: Translation of the Debriefing Assessment for Simulation in Healthcare in Portuguese and cross-cultural adaptation for Portugal and Brazil
Source: Adv Simul (Lond). 2021 Jul 7;6:25. doi: 10.1186/s41077-021-00175-z (PMC8265112; doi:10.1186/s41077-021-00175-z)
Supplement: Supplementary file 3 — Additional file 3. Portuguese Instructor Version. [file 41077_2021_175_MOESM3_ESM.pdf]

DASH - *Debriefing Assessment for Simulation in Healthcare*©
Avaliação do *Debriefing* em Simulação Clínica – Versão do Facilitador

Instruções: Faça uma autoavaliação do seu desempenho na introdução e no *debriefing* desta atividade de simulação. Utilize a seguinte escala para classificar cada um dos seis "Elementos". Para cada Elemento, os Comportamentos são referências para indicar o desempenho positivo no Elemento em questão. Tente classificar a sua **eficácia global para cada Elemento** guiado pelos comportamentos que o caracterizam. Se um dos Comportamentos listados for impossível de avaliar (p. ex. como o facilitador lidou com pessoas descontentes, se ninguém esteve descontente), deixe o espaço em branco e não permita que isso influencie a sua avaliação. Pode ter sido mais, ou menos bem-sucedido dentro de cada Elemento. A classificação do Elemento representa a impressão **geral** do seu desempenho no Elemento em questão. O Elemento 1 avalia a introdução no início da atividade de simulação. Os Elementos 2 a 6 avaliam o *debriefing*.

Escala de Pontuação

Classificação	**1**	**2**	**3**	**4**	**5**	**6**	**7**
Descrição	**Extremamente ineficaz**<br>**Extremamente insatisfatório**	Consistentemente ineficaz<br>Consistentemente insatisfatório	Ineficaz<br>Insatisfatório	Pouco eficaz<br>Satisfatório	Bastante eficaz<br>Bom	Consistentemente eficaz<br>Muito Bom	**Extremamente eficaz**<br>**Excelente**

O Elemento 1 avalia a introdução prévia ao exercício de simulação.
Ignore este elemento se não tiver participado na introdução.

Elemento 1 **Classificação Elemento 1**
Preparei o cenário para uma experiência de aprendizagem envolvente.
____

- Apresentei-me, descrevi o local do cenário e o esperado durante a atividade.
- Apresentei os objetivos da aprendizagem e esclareci questões de confidencialidade.
- Expus os pontos fortes e as limitações da simulação e o que os participantes podem fazer para aproveitar ao máximo a experiência em simulação clínica.
- Informei detalhes logísticos necessários, como localização dos recursos sanitários, disponibilidade de alimentação e programa.
- Estimulei os participantes a compartilhar pensamentos e questões sobre o caso simulado e o *debriefing,* e reforcei que não iriam ser constrangidos ou inferiorizados durante o processo.

Os Elementos 2 a 6 avaliam o *debriefing*

Elemento 2 **Classificação Elemento 2**
Mantive um ambiente envolvente para a aprendizagem.
____

- Esclareci os objetivos do *debriefing*, o que era esperado dos participantes e qual o meu papel no *debriefing*.
- Reconheci os problemas relacionados com o realismo e ajudei os participantes a aprender apesar de se tratarem de casos simulados.
- Mostrei respeito pelos participantes.
- Assegurei que o objetivo principal se centrou na aprendizagem e não em fazer com que os participantes se sentissem mal por cometerem erros.
- Estimulei os participantes a partilharem pensamentos e emoções sem medo de se sentirem constrangidos ou inferiorizados.

Elemento 3 **Classificação Elemento 3**
Estruturei o *debriefing* de forma organizada. __________

- Conduzi a conversa de forma lógica, em vez de saltar de ponto em ponto.
- No início do *debriefing,* incentivei os participantes a partilharem as suas reações genuínas relativamente ao caso e considerei-as de forma séria.
- Durante o *debriefing*, ajudei os participantes a analisarem as suas ações e pensamentos enquanto revíamos o caso.
- No final, foi feito um resumo do caso no qual ajudei os participantes a integrarem todas as observações e **abordei o caso de diferentes maneiras para que eles possam melhorar a sua prática clínica futura.**

Elemento 4 **Classificação Elemento 4**
Promovi discussões que levaram os participantes a refletir sobre o seu desempenho. __________

- Utilizei exemplos concretos – não apenas comentários abstratos e generalizados – para que os participantes pudessem refletir sobre o seu desempenho.
- O meu ponto de vista foi claro; não forcei os participantes a adivinhar o meu pensamento.
- Ouvi e fiz com que os participantes se sentissem ouvidos ao incluir todos na discussão, interpretar os comentários e utilizar linguagem não verbal, como olhar nos olhos dos participantes ou acenar com a cabeça.
- Utilizei vídeos ou dados gravados para apoiar a análise e aprendizagem.
- Se alguém ficou descontente com o *debriefing*, fui respeitador e construtivo ao tentar ajudá-lo a lidar com a situação.

Elemento 5 **Classificação Elemento 5**
Identifiquei o que fizeram melhor e pior – e porquê. __________

- Realizei comentários concretos sobre o desempenho dos participantes e/ou do grupo baseados em declarações objetivas e no meu ponto de vista.
- Ajudei os participantes a explorar o que pensavam ou o que tentavam alcançar nos momentos chave.

Elemento 6 **Classificação Elemento 6**
Ajudei os participantes a perceber como podem melhorar ou manter um bom desempenho. __________

- Ajudei os participantes a aprenderem como podem melhorar deficiências ou como repetir um bom desempenho.
- Usei o meu conhecimento e experiência para ajudar os participantes a perceberem como podem melhorar o seu desempenho no futuro.
- Garanti que todos os pontos importantes foram abordados.

Direitos Autorais. *Center for Medical Simulation*, www.harvardmedsim.org, 2011.
