# Supplementary material for: Translation of the Debriefing Assessment for Simulation in Healthcare in Portuguese and cross-cultural adaptation for Portugal and Brazil
Source: Adv Simul (Lond). 2021 Jul 7;6:25. doi: 10.1186/s41077-021-00175-z (PMC8265112; doi:10.1186/s41077-021-00175-z)
Supplement: Supplementary file 4 — Additional file 4. Portuguese DASH Rater's Handbook. [file 41077_2021_175_MOESM4_ESM.pdf]

Centro para Simulação Médica

____

Avaliação do Debriefing em Simulação Clínica ADSC (DASH)©

Tradução

Centro de Simulação Realística

Instituto Israelita de Ensino e Pesquisa Albert Einstein – Brasil

Centro de Simulação Biomédica

Centro Hospitalar e Universitário de Coimbra – Portugal

____

Manual do Avaliador

____

Introdução

Uma etapa crucial no esclarecimento e na consolidação da aprendizagem com simulação na área da saúde é a realização do *debriefing* – que por definição é rever metodicamente os acontecimentos, e o porquê da sua ocorrência. Conduzir um *debriefing* é uma competência que pode ser aprendida e pode ser melhorada com prática e feedback. O manual de avaliação de *debriefing* em simulação na saúde (DASH) e os formulários de avaliação foram criados para avaliar e ajudar a desenvolver as técnicas de realização de *debriefing*. Informações adicionais, formulários de avaliação e informações de contato podem ser encontradas no site: **http://www.harvardmedsim.org/dash.html**

Histórico do processo de *debriefing*

No contexto de simulação na área da saúde, o *debriefing* é uma conversa entre duas ou mais pessoas para rever um evento simulado ou atividade realizada. No *debriefing*, os participantes podem explorar e analisar as suas ações, linhas de pensamento, estado emocional e outras informações para melhorar o seu desempenho em situações reais. Quando os facilitadores conseguem envolver os participantes há uma melhoria na retenção e aprofundamento dos conhecimentos e isso aumenta a probabilidade do profissional transferir ou reforçar esses conhecimentos, competências e atitudes para sua prática clínica.

Os facilitadores fazem uma comparação implícita entre o nível de desempenho desejado e o nível de desempenho observado durante a simulação. A diferença entre o desempenho desejado e o desempenho realizado é chamada falha de desempenho. Essa diferença pode ser grande, pequena ou não existente, no caso de um evento de desempenho bom ou excelente. Descrever a sua compreensão de falhas de desempenho e investigar possíveis causas para essas dificuldades são ações que constituem um *debriefing* eficaz.

Os facilitadores podem ajudar a melhorar um desempenho insuficiente ou a reforçar um bom desempenho através da exploração e discussão das ações dos participantes. Além disso, eles podem explorar as “perspectivas” – compreensão e premissas dos participantes sobre a realidade apresentada no cenário – que motivam as ações no cenário. Quase todas as ações complexas são direcionadas pelas perspectivas. Os facilitadores podem centrar-se nos momentos, nas ações ou em ambos.

Os profissionais de saúde assumem um risco emocional quando permitem que o seu desempenho seja assistido e analisado pelos seus pares e facilitadores. No entanto, é fundamental desenvolver e manter um ambiente psicologicamente estável para que todos se empenhem na sua participação. Para fazer isso, os facilitadores competentes dão aos participantes o benefício da dúvida (por exemplo, assumir as melhores intenções) e deixam que os erros e bons desempenhos como um mistério que deve ser analisado em oposição a um crime que deve ser punido ou a um sucesso que deve ser louvado.

O feedback do desempenho atual é uma parte crucial do processo de *debriefing*. Os facilitadores competentes não são ríspidos ou negativos nem falsamente contidos ou sem julgamentos. Ao invés disso, eles dão feedback de forma direta e respeitosa e exploram a motivação intrínseca no desempenho do participante. A maioria dos facilitadores deseja melhorar as falhas de desempenho, mas os facilitadores também podem explorar um bom desempenho: quais as ações ou pensamentos ao longo do processo que permitiram que a pessoa e/ou equipa fosse eficaz?

O que é DASH?

O DASH avalia os comportamentos dos facilitadores que facilitam a aprendizagem e a mudança em contextos experimentais. Os comportamentos descritos no DASH derivam de conceitos teóricos relevantes e evidência em pesquisas prévias assim como da observação de centenas de *debriefings* por especialistas.

O DASH foi desenhado para guiar a avaliação de *debriefings* em várias disciplinas e cursos da área da saúde, envolvendo um número diferente de participantes e uma ampla variedade de objetivos educacionais, desde treino de procedimentos como suturar ao desenvolvimento da competência necessária para gerir uma situação de exceção de escala hospitalar. O DASH é também eficaz em várias situações de restrições físicas e de tempo. Pode ser utilizado para avaliar a introdução do curso de simulação (Elemento 1) e o *debriefing*

pós-simulação (Elementos 2-6). O sucesso do facilitador a criar um ambiente propício para a aprendizagem efetiva afeta as atividades subsequentes; pontuar a introdução irá enriquecer o significado da pontuação total.
O DASH segue e classifica seis elementos principais do *debriefing*. Estes incluem se e como o facilitador:
(1) Estabelece um ambiente envolvente de aprendizagem;
(2) Mantém um ambiente envolvente de aprendizagem;
(3) Estrutura o *debriefing* de forma organizada;
(4) Estimula discussões;
(5) Identifica e explora as falhas de desempenho; e
(6) Ajuda os participantes a alcançarem ou manterem um bom desempenho futuro.
O DASH é uma escala de classificação ancorada em comportamentos, o que significa que é baseada nos comportamentos necessários para executar um *debriefing* eficaz, assim como características de um *debriefing* fraco. Um "Elemento" no DASH é um conceito estrutural que descreve um conjunto de comportamentos do facilitador. Cada "Elemento" é composto por "Dimensões" que refletem partes das competências estruturais. Os "Comportamentos" marcados dentro de cada Dimensão são exemplos concretos da realização desses elementos. Os comportamentos listados são exemplos, mas não estão limitados somente a essa lista.

Orientações para pontuação
Pontuar os facilitadores em cada Elemento usando as descrições de dimensão e exemplos de comportamentos como guias. Uma pontuação completa do DASH inclui cinco elementos de pontuação (ou seis, se o elemento 1, que é a introdução do curso, for observado). As avaliações são baseadas numa escala de 07 pontos de efetividade.

Escala de Avaliação

Escala	Descritor
7	Extremamente eficaz / Excelente
6	Consistentemente eficaz / Muito bom
5	Bastante eficaz / Bom
4	Pouco eficaz / Satisfatório
3	Ineficaz / Insatisfatório
2	Consistentemente ineficaz / Consistentemente insatisfatório
1	Extremamente ineficaz / Extremamente insatisfatório

Como avaliador, deve estudar os Elementos e estar completamente familiarizado com estes. Sintetizar a impressão geral de eficácia do elemento todo, guiado pela observação das dimensões individuais e comportamentos que as definem. Pense de forma holística e não aritmética para considerar o impacto cumulativo das dimensões, que podem não ter o mesmo peso. O avaliador, irá ponderar as dimensões como achar mais adequado e enquadrado, baseado na sua visão holística do Elemento avaliado. Se não for possível avaliar uma dimensão, não deixe que isso influencie a sua avaliação. Por exemplo, se nenhum participante ficar descontente, basta desconsiderar a dimensão em que o facilitador lida com participantes que ficam descontentes ao longo do *debriefing*.
Quando os avaliadores se tornam completamente familiarizados com os Elementos estes referem que a escala é fácil de usar.Os avaliadores experientes estão habilitados a produzir avaliações válidas e confiáveis.
É esperado que *debriefings* curtos e focados em ações e não na compreensão do participante do que aconteceu no cenário sejam pontuados no máximo com scores de 4 ou 5, ou ocasionalmente 6. Deve-se compreender que a condição do *debriefing* pode afetar as classificações. Se a envolvência do debriefing não for ideal – uma sala pequena que limite as distrações do mundo externo ou o mesmo espaço onde ocorreu a simulação – com apenas alguns minutos para poupar, é razoável ver esperar que as melhores notas atinjam somente o nível "Satisfatório". Quando os participantes sentem pressão de tempo, falta de privacidade ou estão muito próximos de uma situação clínica real ou distrações clínicas, é difícil de aprofundar a análise do pensamento na base do comportamento (as causas ou linhas de pensamentos para ações realizadas).
Há uma sobreposição proposital entre os Elementos. Por exemplo, Elemento 1, "Estabelecer um ambiente envolvente de aprendizagem", contém algumas das Dimensões do Elemento 2 "Mantém um ambiente envolvente de aprendizagem". Em todos os casos e para todos os Elementos, os avaliadores devem ignorar a sobreposição e avaliar cada elemento de forma independente dos outros.

Formulários de Avaliação

Há dois formulários de avaliação disponíveis para avaliadores. O formulário curto, desenhado para obter somente a nota do Elemento, é apropriado especialmente para avaliações sumativas. O formulário longo foi desenhado para obter as notas das dimensões e elementos e é especialmente útil para realizar um *feedback* formativo para os avaliadores.

Elemento 1

ESTABELECE UM AMBIENTE DE APRENDIZAGEM ENVOLVENTE

A qualidade do facilitador na introdução da simulação clínica pode definir o tom para tudo o que se segue. Antes de qualquer simulação começar, o facilitador ajuda a esclarecer aos participantes, o que é esperado deles e ajuda-os a entenderem os benefícios e limites de um ambiente de simulação clínica. O facilitador informa os participantes se e como o caso, evento ou procedimento será discutido depois (por exemplo: discutido e analisado) e se a simulação será gravada. O facilitador faz um plano que é focado na aprendizagem e não em "apanhar" erros, e cria um ambiente no qual os participantes se sentem seguros em partilhar as suas ideias e sentimentos sobre a simulação e o debriefing, sem medo de serem humilhados.

Elemento 1 – DIMENSÕES

Esclarece o objetivo do curso, ambiente, papéis e expectativas.
Os cursos baseados em simulação fluem melhor e os participantes ficam mais envolvidos quando entendem o motivo de estar a realizar determinado cenário, o papel deles e dos facilitadores e o que é esperado de cada participante.

Comportamentos eficazes e positivos para essa dimensão incluem:
- Apresentar-se e convidar os demais a apresentarem-se.
- Compartilhar e convidar os demais a partilhar informações sobre suas qualificações, experiências pessoais e interesses no curso.
- Apresentar uma visão geral do curso e dos objetivos de aprendizagem.
- Introduzir e descrever simuladores, acessórios, localização dos equipamentos, papel dos atores, etc.
- Alinhar expectativas para que os participantes se comprometam em utilizar os equipamentos disponíveis (por exemplo, realizar os procedimentos como na realidade, tomando cuidado com o paciente simulado).
- Discutir a base dos casos, procedimentos ou eventos a serem simulados e porque certos casos ou tarefas foram incluídos no curso (por exemplo, casos reais que tiveram resultados desfavoráveis que são particularmente difíceis de praticar, etc.).
- Explicitar as expectativas para os papéis dos participantes durante a simulação e *debriefing*.
- Pedir aos participantes envolvimento nas discussões no *debriefing* e que tentem fazer uma autorreflexão.
- Afirmar quais são as regras de etiqueta para o *debriefing*: ser respeitoso, curioso e educado sobre os pensamentos e ações dos demais.
- Encorajar as pessoas a falarem ou permitir discordar respeitosamente.
- Descrever explicitamente o papel do facilitador: facilitar a discussão; comentar o desempenho baseando-se na observação de eventos similares ou simulações; atuando como um próprio recurso em sua área de especialização (por exemplo, PALS, ACLS, CRM, trabalho em equipa, clínica); e garantindo que os objetivos do treino foram alcançados.

Comportamentos negativos e ineficazes incluem:

- Não descrever objetivos, papéis ou expectativas.
- Ser vago ou descrever de forma enganosa a simulação ou o *debriefing*.
- Não deixar tempo ou oportunidade para as questões dos participantes.

Estabelecer um "contrato de ficção" com os participantes
O contrato de ficção é um entendimento entre o facilitador e o participante. Nele, o facilitador reconhece que a simulação não pode ser exatamente como a vida real, mas concorda em fazer a simulação com o maior realismo possível, dentro das limitações dos recursos e tecnologias. Os participantes concordam em fazer o melhor para agir como se tudo fosse real.

Comportamentos positivos e eficazes para essa dimensão incluem:
- Explicar que o facilitador e participantes devem colaborar para criar uma simulação envolvente e um ambiente de aprendizagem.
- Afirmar que a obrigação do facilitador é fazer tudo que torne a simulação o mais real possível dentro das limitações dos recursos e das tecnologias.
- Solicitar aos participantes para atuarem o melhor que puderem, como se a simulação fosse real, reconhecendo que um participante irá atuar de forma diferente num ambiente de simulação e num ambiente clínico real.
- Realizar uma avaliação justa e equilibrada dos pontos fortes e dos pontos fracos dos simuladores.

Comportamentos negativos ou ineficazes incluem:
- Trivializar os desafios enfrentados pelos participantes em integrar o realismo da simulação.
- Afirmar ou assumir que os participantes deveriam ou irão atuar da mesma forma que eles agem num ambiente clínico real.
- Insinuar ou afirmar que a culpa é do participante se a simulação não lhe parece real.

Detalhes logísticos
Apesar de parecer secundário, informar os participantes sobre detalhes logísticos e promover um ambiente físico confortável ajuda os participantes a focarem-se na aprendizagem.

Comportamentos positivos e eficazes para essa dimensão incluem:
- Ter certeza de que o espaço para simulação está limpo. Quando disponível, cadeiras, mesas, quadro, vídeo, dispositivos de simulação ou outros materiais educacionais devem estar em ordem e limpos, prontos para quando os participantes chegarem.
- Instruir participantes sobre o local onde acontecerá a simulação e quanto tempo irá durar.

- Informar os participantes sobre a disponibilidade de comida e bebida, transportes e outras considerações logísticas, localização dos sanitários, etc.
- Informar aos participantes sobre quando e onde será o caso simulado, procedimento ou evento será discutido.

Comportamentos negativos ou ineficazes incluem:
- Não orientar os participantes sobre a logística do curso e do ambiente físico.
- Ignorar ou menosprezar as eventuais preocupações dos participantes sobre tempo, localização e necessidades físicas.
- Não abordar potenciais desafios dos indivíduos relacionados às condições físicas (por exemplo, se utilizam cadeiras de rodas ou outros dispositivos, se tem alergia ao látex, etc.)

Transmite o compromisso de respeitar os participantes e entender a perspectiva deles
Os participantes preocupam-se frequentemente com o facto das simulações serem desenhadas para expor suas fraquezas ou para humilhá-los.

Para combater esses conceitos, os facilitadores devem transmitir que assumem que o participante tem boas intenções e que estará a dar o seu melhor, mas que provavelmente cometerá erros ao longo do caminho – o que é perfeitamente normal, pois este é um bom lugar para conversar sobre como melhorar a nossa prática.

Comportamentos positivos e eficazes nessa dimensão incluem:
- Afirmar algo como "Erros são como enigmas que devem ser resolvidos e não crimes que devem ser punidos".
- Afirmar que ele/ela entende que o participante está tentando alcançar algo positivo até quando comete erros.
- Expressar algo como "pressupostos básicos" que os participantes são inteligentes, bem-treinados e que estão a fazer o melhor para aprender e melhorar.

Comportamentos negativos e ineficazes incluem:
- Provocar, desprezar ou ignorar as expressões de ansiedade.
- Afirmar ou deixar implícito que o desempenho fraco apresentado pelos participantes será usado contra eles.
- Fazer comentários degradantes sobre a competência dos participantes.
- Dizer coisas que prejudiquem a aspiração de um participante em ser um bom profissional da saúde.

Elemento 2

MANTÉM UM AMBIENTE DE APRENDIZAGEM ENVOLVENTE.
Este elemento sobrepõe-se claramente ao Elemento 1 e engloba a capacidade do facilitador manter um bom ambiente de aprendizagem. No início do debriefing, especialmente no primeiro do dia, e ao longo do debriefing, o facilitador deve ajudar a esclarecer os participantes sobre o que é esperado deles. O facilitador deve garantir que os participantes se sentem confiantes em relação à segurança do ambiente para partilhar os seus pensamentos e sentimentos – isto é, eles não se devem sentir envergonhados ou humilhados – e que o foco é na aprendizagem e não em "apanhar" o erro de alguém.

Elemento 2 – Dimensões

Esclarece os objetivos, papéis e expectativas do *debriefing*
O *debriefing* flui melhor e os participantes ficam mais envolvidos quando entendem o que estão a fazer, o seu papel e o papel do facilitador e o que é esperado deles.

Comportamentos positivos e eficazes incluem:
- Esclarece os tópicos que serão abordados (por exemplo, "Eu gostaria de discutir como se identificam arritmias.")
- Esclarece o papel dos participantes durante o *debriefing*.
- Solicita que os participantes colaborem nas discussões e tentem fazer uma autorreflexão.
- Descreve o papel do facilitador explicitamente: facilitar a discussão, comentar o desempenho observado na simulação e atuar como um especialista na sua área de experiência (por exemplo, PALS, ACLS, CRM, trabalho em equipa ou clínica); para assegurar que os objetivos do treino sejam alcançados.

Comportamentos negativos e ineficazes incluem:
- Não falar sobre os papéis do facilitador ou dos participantes.
- Deixar vagos ou não declarar os objetivos do *debriefing*.
- Não dar tempo para perguntas ou comentários dos participantes.
- Não valorizar às expressões verbais e não verbais feitas pelos participantes que indicam que eles estão confusos com os objetivos.

Ajudar os participantes a envolverem-se num contexto de realismo limitado
Frequentemente os participantes não apreciam (ou ressentem) as limitações do realismo das simulações até passarem por uma simulação específica. Eles querem ter um bom desempenho e sentem-se mal quando isso não acontece. Se os participantes se sentirem enganados ou frustrados pela tecnologia de simulação ou se preocuparem em não serem vistos de forma competente, vão ficar insatisfeitos. Às vezes ficam simplesmente frustrados quando a limitação do realismo confunde o processo de resolução de problemas. Os facilitadores podem ajudar os participantes a abordar essas questões, reconhecendo a legitimidade de suas queixas, mas mantendo o *debriefing* focado nos

objetivos de aprendizagem e não no equipamento. Os facilitadores reforçam a seriedade e o realismo do cenário ou procedimentos simulados, tratando-os como casos reais e tratando os manequins, pacientes simulados/atores profissionais como se fossem pessoas com integridade e motivações reais. Por exemplo, se o ator está a simular um médico que está agressivo, a gritar, o facilitador deve ajudar os participantes a explorar quais são os reais problemas daquele personagem.

Comportamentos bons e eficazes incluem:
- Reforçar aos participantes que as partes físicas do simulador são diferentes da vida real.
- Reforçar que os participantes podem agir de forma um pouco diferente num ambiente simulado.
- Reforçar ou demonstrar que apesar das limitações da simulação, ainda assim é um recurso útil para discussão (por exemplo: comparando-se os eventos simulados com situações da vida real).
- Reconhecer os problemas ou reclamações dos participantes parafraseando e aceitando essas queixas. (Por exemplo: "Está frustrado com os sons respiratórios. Sim, eles não são ótimos.").
- Após validar a experiência dos participantes, direcionar a discussão para pontos produtivos (por exemplo: "É verdade, pode ser difícil discernir os sons respiratórios num simulador e eu compreendo isso". Eu gostaria de saber se alguém, alguma vez teve dificuldades de identificar os sons respiratórios na vida real e como se sentiu em relação a isso?)
- Reconhecer que há certa estranheza em "simular".

Comportamentos negativos e ineficazes incluem:
- Discutir com o participante o realismo do cenário;
- Negar a legitimidade das queixas dos participantes;
- Mostrar ressentimento pelos participantes não se terem envolvido completamente na simulação;
- Rir ou depreciar a tecnologia ou atores utilizados na simulação;
- Fazer com que os participantes se sintam ridicularizados por terem se dedicado plenamente ao caso, procedimento ou evento apresentado.

Transmitir respeito pelos participantes e preocupar-se com a sua segurança psicológica
Transmitir respeito e realizar considerações positivas aos participantes ajuda a criar um contexto de conversa propício à discussão crítica, necessário para um bom *debriefing*. Os facilitadores criam um ambiente favorável quando tratam os participantes como membros de uma equipa respeitada levando em consideração as suas categorias profissionais. Demonstrar esse respeito pode andar de mãos dadas com críticas pontuais ao desempenho do participante. Não é necessário esconder julgamentos, mas é necessário realizar as questões e respeitosamente ouvir as respostas.

Comportamentos bons e eficazes incluem:
- Demonstrar curiosidade genuína incentivando os participantes a partilharem os seus pensamentos e a ouvir as suas respostas;
- Realizar perguntas abertas que encorajem os participantes a explorar as suas ideias;
- Realizar anotações ou lembretes de comentários feitos pelos participantes e estabelecer conexões ao longo do *debriefing*;
- Criticar os comportamentos e não as pessoas (por exemplo, dizendo, "Gritar com a enfermeira não fez com que tivesse as respostas que precisava", ao invés de, "Você é nervoso, não é?").

Comportamentos negativos e ineficazes incluem:
- Perguntar e responder às suas próprias perguntas;
- Falar por cima das observações dos participantes;
- Humilhar os participantes ou ridicularizar as suas respostas;
- Seguir uma linha de questões com a intenção de fazer o participante parecer ou sentir-se incapaz;
- Usar sarcasmo ou ironia nas discussões sobre as ações ou pensamentos dos participantes.

Elemento 3

ESTRUTURA O *DEBRIEFING* DE FORMA ORGANIZADA
Enquanto diferentes escolas de debriefing têm números diferentes de fases, quase todas as abordagens advogam em seguir um fluxo lógico que permita que os participantes: 1) Exponham as reações imediatas; 2) analisem o que aconteceu e generalizem ou apliquem para prática atual ou futura; e 3) resumam a aprendizagem. Assim sendo, um debriefing eficaz deveria começar pelos participantes a expor as suas reações iniciais; o meio do debriefing deveria compreender uma fase de entendimento que inclui a análise e generalização das outras conformações e o debriefing deveria ser concluído com uma frase de resumo.

Elemento 3 – Dimensões

Encoraja os participantes a expressarem as suas reações e, se necessário, orienta-os para o que aconteceu na simulação, logo no início.
Essa fase inicial do *debriefing* permite que os participantes expressem suas emoções iniciais em relação à simulação e, se necessário, que o facilitador forneça informações que esclareçam os fatos subjacentes à simulação. As questões do facilitador e as respostas ao que os participantes dizem, podem ajudar ou dificultar o desenvolvimento da segurança psicológica. Convidar os participantes a demonstrar as suas reações e ouvir com interesse ajuda a criar uma sensação de segurança no *debriefing*. O facilitador certifica-se que os participantes entenderam o caso, procedimento ou evento e pode esclarecer fatos que não foram compreendidos. Os comentários dos participantes fornecem dicas sobre as suas preocupações e informam o facilitador sobre quais os objetivos importantes a reforçar.

Comportamentos positivos e eficazes incluem:

- Realizar perguntas que convidam os participantes a expressar as ideias iniciais ao caso, procedimento ou evento, e permitir que emoções sejam expressas;
- Aceitar e encorajar reações balançando a cabeça e parafraseando o participante;
- Discutir os fatos e rever os eventos quando necessário para eliminar confusões expressas pelos participantes. Isso também pode ser realizado na fase de análise.

Comportamentos negativos e ineficazes incluem:
- Ridicularizar as respostas dos participantes à experiência;
- Tornar-se defensivo sobre as reações negativas que os participantes podem expressar sobre o caso, procedimento ou evento.
- Saltar a fase de reações, o que pode levar o participante sentir-se inseguro.

Guiar a análise do desempenho dos participantes durante o meio da sessão.
O propósito da fase de análise é permitir que os eventos da simulação façam sentido aos participantes, que eles possam abordar as suas preocupações e isso os ajude a atingir os objetivos do curso. Os facilitadores devem-se focar em explicar os pensamentos do processo e os sentimentos que dirigiram as ações dos participantes, e então, trabalhar com os participantes para que repensem ou aumentem esses guias de desempenho para garantir um melhor cuidado ao paciente em situações similares no futuro. Esta fase do *debriefing*, também pode ajudar os participantes a refletir sobre como as simulações podem ser generalizadas a outras situações.

Comportamentos positivos e eficazes incluem:
- Fazer perguntas que façam o participante discutir e refletir sobre o que ele pensou, sentiu e o que aconteceu durante a simulação.
- Ouvir as questões levantadas pelos participantes de forma a que o facilitador possa adaptar palestras curtas para responder às dúvidas dos participantes.
- Gastar tempo apropriado em tópicos de grande interesse para os participantes e ao mesmo tempo cobrir os tópicos que fazem parte do que é requerido pelo curso.
- Fazer questões e incentivar discussões que levam os participantes a especularem como as lições aprendidas na simulação podem ser aplicadas no ambiente de assistência ao paciente – em outras palavras, ajudar os participantes a generalizar a partir da experiência na simulação.

Comportamentos negativos e ineficazes incluem:
- Dizer ao participante de forma imediata o que fazer diferente numa próxima vez (Por exemplo; "Chame ajuda imediatamente: não espere!") sem estimular o raciocínio sobre o que e porque fizeram determinada ação.
- Realizar *debriefing* através de uma lista de objetivos de aprendizagem sem considerar ações específicas da perspectiva do participante – positivas ou negativas – ao longo do caso, procedimento ou evento.
- Interromper as explicações dos participantes dos motivos que os levaram a realizar determinada linha de ações.

Colaborar com os participantes para que resumam a aprendizagem da sessão de *debriefing*, próximo ao final.

O propósito da fase de resumo é sinalizar o final do *debriefing*, rever pontos importantes e traduzir as lições aprendidas com o *debriefing*, dentro de princípios memoráveis que participantes levam com eles para melhorar a sua prática.

Comportamentos positivos e eficazes incluem:
- Esclarecer que esse momento é destinado ao encerramento das discussões e para que os participantes façam um resumo das lições aprendidas;
- Fazer uma ou várias perguntas aos participantes que os ajude a resumir o que foi aprendido (Por exemplo, "O que foi realizado bem"? Em circunstâncias similares, o que fariam diferente em uma próxima vez? Que lições podem usar na vossa prática diária?)
- Resumir pontos importantes que não foram referidos pelos participantes.
- Recomendar leituras ou atividades que os participantes podem realizar para melhorar o seu desempenho.

Comportamentos negativos e ineficazes incluem:
- Terminar o *debriefing* de forma abrupta sem resumir os principais pontos de aprendizado (Por exemplo, "Ah, olhem a hora! Isso é tudo o que temos tempo para hoje. Tenham um bom dia").
- Monopolizar as discussões finais sem dar oportunidade aos participantes para concluírem suas observações.
- Tratar o *debriefing* como uma experiência isolada sem enfatizar a sua conexão com a experiência prévia dos participantes e suas práticas futuras.

Elemento 4

Motiva discussões envolventes

Qual capacidade do facilitador envolver os participantes em discussões interessantes, ouvir o que eles dizem e ajudá-los a serem profissionais que refletem? O objetivo do debriefing é conseguir que participantes se foquem em tópicos importantes e gerar discussões profundas. O debriefing não deveria ser focado somente em levantar fatos e conhecimentos. Ao invés disso, bons debriefings exigem que os participantes apliquem, analisem, resumam e avaliem informações. O objetivo do debriefing é encorajar participantes a refletirem pessoalmente na sua prática clínica ou gestão em saúde e inspirar melhorias.

Dimensões Elemento 4
Usa exemplos concretos e desfechos como base das suas questões e discussão.

Examinar as ações clínicas, sociais ou de trabalho em equipa tomadas pelos participantes e o desfecho do caso, procedimento ou evento. Permite que o facilitador e participante trabalhem com dados verificáveis e públicos como ponto inicial da discussão. Isso pode incluir, por exemplo, se o paciente parou ou se informações importantes foram perdidas. A partir desse ponto, os facilitadores exploram as "perspectivas" pessoais do participante que motivaram as suas ações.

Comportamentos positivos e eficazes incluem:

- Fazer perguntas baseadas em ações ou resultados observados. "Pareceu-me que demorou 3 minutos para a desfibrilação do paciente"...
- Usar observações, comportamentos e interações para provocar discussões sobre processos mentais ou maneiras de melhorar ações no futuro. "Pareceu-me que demorou 3 minutos para a desfibrillação do paciente. No *debriefing* anterior, discutimos a importância de chocar um paciente rapidamente. Isso me faz pensar que pode existir alguma dificuldade em desempenhar essa tarefa. Como vocês veem isso?"

Comportamentos negativos e ineficazes incluem:

- Fazer deduções sobre o desempenho do participante (ex. "você não sabia qual era o ritmo") sem reconhecer que são deduções e desse modo sujeitando-se a correções dos participantes
- Insistir no desfecho do caso, procedimento ou evento, particularmente quando negativos, sem discutir a sequência de ações na análise.

Revela o seu raciocínio e julgamentos.
Existe uma longa tradição de questões e diálogos socráticos na educação em saúde. Muitas vezes esse processo é executado de modo que fere o espírito e a letra do diálogo socrático, um método em que tanto os processos mentais do professor como os dos participantes deveriam estar abertos à discussão. Ao invés disso, facilitadores para profissionais de saúde muitas vezes enunciam uma série de perguntas sem revelar seu raciocínio. O resultado de esconder um raciocínio é que os participantes frequentemente ficam confusos em relação ao porque uma pergunta foi feita; ou pior, podem sentir-se manipulados ou numa armadilha. Os facilitadores podem evitar esses problemas revelando o seu raciocínio para seguir uma linha de perguntas, fazendo isso de maneira curiosa e respeitosa para com o participante. Idealmente, suposições ou conclusões que um facilitador fez sobre o desempenho dos participantes são comunicados de forma a serem abertos à discussão pelos próprios praticantes. Ou seja, os facilitadores devem ver as suas próprias conclusões com ceticismo e assumir que o participante é inteligente e bem-intencionado.

Comportamentos positivos e eficazes incluem:

- Descrever o seu próprio raciocínio para deixar claro que o assunto é importante para o *debriefing*. Ex. "Mesmo que a compressão torácica pareça ser algo simples de fazer, muitas vezes as pessoas têm problemas durante a ressuscitação cardiopulmonar. Assim gostaria de discutir os desafios e truques para manter o ritmo e permitir o retorno do tórax".
- Fazer afirmações respeitosas sobre julgamentos pessoais ou preocupações sobre desempenho (bom, mau, pouco usual, interessante, alarmante, etc.) de maneira a que o participante não tenha que adivinhar porque é questionado ou fique confuso quanto ao ponto de vista do facilitador. Ex. "Eu não o vi fazer verificação da dose da medicação. Sem uma dupla verificação, eu fico preocupado, pois existe uma maior probabilidade de ser administrada uma dose errada ao paciente. Eu queria perceber qual era o seu pensamento naquele momento"?
- Fornecer informações que suportem o ponto de vista do facilitador, como experiência pessoal, observação de outros, literatura, etc.
- Expressar inferências ou crenças sobre o desempenho do participante em termos relativos (ex. ao invés de dizer "Não havia um líder", dizer "pareceu-me que vocês tiveram problemas para designar um líder neste caso") e não assumir saber o que os participantes estavam pensando no momento.

Comportamentos negativos e ineficazes incluem:

- Esconder as suas preocupações ou seu raciocínio.
- Fazer perguntas direcionadas que contenham julgamentos ou soluções. (ex. "Não seria melhor ter chamado ajuda mais cedo?)".
- Fazer perguntas que forcem participantes a demonstrar falta de conhecimento ou admitir erros sem revelar o propósito da questão. (ex. "quando é que o beta bloqueador está indicado?)".
- Afirmar as suas próprias conclusões como verdade absoluta. Ex. "Estava fixado. Não considerou a anafilaxia como possível diagnóstico".

Facilita discussão através de técnicas verbais e não verbais
Os facilitadores utilizam técnicas verbais e não verbais tanto de forma consciente como inconsciente durante o *debriefing*. Este elemento está relacionado com o uso dessas técnicas para conseguir opiniões e controlar a discussão.

Comportamentos positivos e eficazes incluem:

- Envolver toda a equipa; não permitir que uma ou duas pessoas dominem a discussão.
- Evocar e utilizar diferentes pontos de vista para enriquecer a compreensão de um tópico. "Então, Francisco parece que acha que o fato do problema do paciente ser iatrogénico não deveria influenciar a adesão a diretivas antecipadas de vontade. Mas a opinião da Helena é que isso deveria ser levado em consideração. Vocês podem ajudar-me a entender o raciocínio que justifica estas posições?"
- Ajuda a trazer pessoas que estão quietas para a discussão utilizando questões reais sobre o cenário. Ex. "Raul, eu reparei que reviu o prontuário do paciente, o que chamou a sua atenção"?
- Ouve calmamente comentários dos participantes sem os interromper.
- Usa linguagem corporal como acenar com a cabeça, contato visual, postura, proximidade e distância, levantar ou sentar, expressão facial para ajudar nas questões, ou para demonstrar interesse, gentileza ou poder, com a finalidade de fomentar uma discussão produtiva.
- Usa o silêncio de forma a dar tempo aos participantes para pensarem sobre questões levantadas.

- Repete por outras palavras verbalmente o que participantes dizem.

Comportamentos negativos e ineficazes incluem:
- Franzir a testa, desviar olhar, bater os dedos ou aparentar irritação ou inquietação.
- Não dar tempo de silêncio para os participantes poderem manifestar a sua posição.
- Resmungar durante o discurso dos outros.
- Interromper ou cortar a voz aos participantes de forma abrupta.
- Permitir que alguns participantes dominem a discussão ou favorecer algum participante demonstrando maior interesse no que ele diz.
- Consistentemente permitir que alguns participantes sejam excluídos da conversa.

Usa vídeo, *replay* ou outros dispositivos para revisão (se disponíveis).
Vídeos ou outros mecanismos para rever o caso são técnicas educacionais efetivas e podem ser utilizadas para ajudar os participantes a ver o seu desempenho e relacioná-lo com os objetivos chave do *debriefing*.

Comportamentos positivos e eficazes incluem:
- Mostrar um ou poucos segmentos curtos para ilustrar ou introduzir tópicos.
- Usar a revisão como ponto de partida para discussão.
- Operar o equipamento de forma eficiente (ex. encontra segmentos desejados rapidamente).
- Associa imagens com objetivos chave; usa dispositivos de vídeo e revisão para focar algo importante ou para aprofundar a discussão.
- Pausa a revisão do segmento se discussão substancial ocorrer.

Comportamentos negativos e ineficazes incluem:
- Passar longos trechos de vídeo sem nenhuma discussão ou sem um propósito específico.
- Não utilizar o vídeo quando esse poderia ser útil para realçar pontos importantes ou para resolver um conflito.
- Realizar comentários impróprios sobre a aparência ou a voz de alguém no vídeo.

Reconhece e aborda participantes descontentes.
Não valorize esta dimensão se nenhum participante aparenta estar insatisfeito.

No melhor momento, a simulação é emocionalmente mobilizadora. Assim, há naturalmente momentos em que os participantes ficam insatisfeitos. O facilitador habilidoso ajudará o participante a aliviar o ambiente e a ajudar o grupo a voltar a um estado emocional estável. Mesmo um participante moderadamente descontente permite valorizar essa dimensão.

Comportamentos positivos e eficazes incluem:
- Perceber e reconhecer quando alguém está insatisfeito, salientar essa observação e respeitosamente confirmar com o participante se essa observação é correta.
- Convidar ou permitir que o participante descreva os seus sentimentos – se ele assim quiser.
- Tentar uma variedade de técnicas para reestabelecer o equilíbrio, como normalizar o comportamento (ex. se um participante está insatisfeito sobre seu desempenho, colocar no contexto do desempenho de outros participantes em simulações semelhantes, dizendo algo como; "já fizemos esse cenário 40 vezes e quase todos lidam com o caso tal como o fez"), permitir que outros participantes defendam ou apoiem o colega, etc.
- Lidar de forma aberta com a causa do descontentamento.
- Guiar a discussão e tempo para ajudar o participante a confrontar e resolver a causa do descontentamento, possivelmente mudando o ritmo da discussão; distribuindo a conversa para outros explorarem a sua perspectiva ou contribuírem para o assunto perturbador ao invés de focar exclusivamente no participante descontente; recuar para assuntos menos emocionais, mas voltar ao assunto difícil seja durante o *debriefing* ou depois de forma privada com o participante.

Comportamentos negativos e ineficazes incluem:
- Ignorar, perseguir ou condenar ao ostracismo.
- Rir ou diminuir a causa da insatisfação da pessoa.
- Criar rivalidades entre os participantes sobre assuntos em que discordaram.

Elemento 5

Identifica e explora falhas de desempenho
Ao avaliar esse Elemento, considerar a capacidade do facilitador descrever falhas de desempenho – isto é, a diferença entre o desempenho atual e um desempenho ótimo. Os facilitadores devem dar aos participantes um parecer concreto sobre o seu desempenho. Além de identificar, o facilitador deve explorar as causas dessa falha. Quando um desempenho é subótimo, o facilitador trabalha com o participante para analisar como as "perspectivas" (incluindo conhecimentos e atitudes) e habilidades do participante levaram à falha de desempenho. Se um participante teve uma boa prestação, não significa que não exista nada a discutir: na situação de desempenho bom ou excelente, o facilitador auxilia o participante a identificar os conhecimentos, habilidades e atitudes que levaram a esse bom desempenho.

Dimensões Elemento 5
Fornece um parecer sobre o desempenho.
Quando estão a aprender uma competência complexa, os participantes, por vezes, não têm uma noção clara de como estão a progredir, (tal como uma pessoa que aprende a conduzir pode ter problemas para perceber que está na faixa certa). Os participantes precisam de comentários, e isso habitualmente significa realçar a distância que existe entre onde eles estão e onde querem ir. Um parecer claro –

apreciação crítica – sobre como o desempenho dos participantes está aquém, adequado ou além das expectativas para essa atividade de simulação e é crucial para a aprendizagem.

Comportamentos positivos e eficazes incluem:
- Expor críticas ao desempenho de participantes de forma específica e ligada a ações. (Ex. ao invés de dizer "a comunicação foi má", dizer "Eu não o vi olhar nos olhos da paciente ou parafrasear o que ela disse)".
- Comentar o desempenho positivo e não apenas o negativo.
- Ser direto, porém respeitoso. (Ex. "Eu não o ouvi pedir desculpa ao paciente pelo erro. Penso que isso contribuiu para ele ficar irritado"; ex. "Eu não o vi fazer dupla verificação. Isso preocupa-me, pois aumenta o risco de um erro de medicação)".

Comportamentos negativos e ineficazes incluem:
- Falar em revelar opinião ou julgamento quando descreve observações sobre desempenho dos participantes
- Disfarçar críticas embelezando, camuflando ou usando a "técnica da sanduíche" (afirmação positiva seguida de criticismo seguida de afirmação positiva).
- Fazer perguntas tendenciosas de forma a que os participantes exponham a falha de desempenho que o facilitador observou, mas não quer dizer.
- Expressar críticas em tom sarcástico, nocivo, ofensivo ou desrespeitoso (ex. "não me admira que o paciente tenha começado a gritar! Que parte de "desculpa" é tão difícil de dizer?!)".

Explora a base da falha de desempenho
Explorar o motivo ou raciocínio para os participantes estarem onde eles estão ao invés de onde poderiam estar é uma característica relevante a um bom *debriefing*. É útil ajudar os participantes a entender como a suas perspectivas (como conhecimentos, premissas, crenças ou sentimentos) contribuem para o seu desempenho. Os facilitadores devem ajudar os participantes a explorar os fundamentos das suas ações. Ocasionalmente, é necessário, por limites de tempo ou pelo objetivo do curso, focar somente nas ações corretas ao invés de ajudar os participantes a explorar e repensar suas perspectivas. Um *debriefing* que foca exclusivamente nas ações geralmente não será classificado acima de 4 e raramente receberá uma nota 5 nesse elemento.

Comportamentos positivos e eficazes incluem:
- Ir além do "o quê" para o "porquê", isto é, explorar porque os participantes tomaram as ações sem focar exclusivamente os prós e contras de cada ação.
- Encorajar os participantes a refletir no que estavam a pensar durante o cenário.
- Fornecer críticas específicas e ligadas a ações. Ex. "Eu não o ouvi resumir as preocupações da mãe". Ou "Estava com os seus ombros encolhidos – consegue ver no vídeo? - penso que isso pode ter contribuído para a fadiga e desconforto que sentiu durante a laparoscopia. Tente baixar os ombros na próxima vez".
- Quando apropriado ao objetivo do curso, ajudar o participante a explorar e entender questões comportamentais que podem contribuir para falhas de desempenho, como estilo de comunicação, planeamento, gestão de carga de trabalho, etc.
- Quando apropriado ao curso, ajudar o participante a entender as questões sinestésicas ou psicomotoras que contribuíram para falhas de desempenho, como profundidade inadequada de compressões ou técnica inadequada de sutura durante uma anastomose intestinal.
- Quando apropriado ao curso, ajudar o participante a entender como o seu conhecimento clínico, premissas e sua aplicação contribuíram para falhas de desempenho, como causas de atraso no diagnóstico e tratamento de embolia gasosa.

Comportamentos negativos e ineficazes incluem:
- Focar apenas na correção das ações.
- Interromper a discussão e processos de pensamento do participante.
- Utilizar caracterizações superficiais ou muito abstratas de comportamentos (ex. "Não ouviu a mãe do paciente", ex. "Precisa de relaxar mais os ombros, assim não se cansa durante o procedimento)".
- Desdenhar hipóteses, conhecimento inadequado ou coordenação física dos participantes.

Elemento 6

Ajuda os participantes a obter ou sustentar um bom desempenho futuro
Avaliar este Elemento requer medir a capacidade efetiva do facilitador em ajudar os participantes a alinharem o desempenho hipotético futuro com expectativas ou repetir um desempenho bom ou excelente.
Os debriefings devem auxiliar os participantes a desenvolver conhecimentos, habilidades e atitudes para eliminar as falhas entre o nível de desempenho que o facilitador deseja e o que foi observado. No caso do desempenho ser bom ou excelente, o facilitador deverá identificar os comportamentos ou respostas que o tornaram especialmente bem sucedido e descobrir os processos mentais ocultos que o permitiram.
Ajudar os participantes a perceberem que ações ou perspectivas funcionam bem e ajudam a sustentar um bom desempenho. O facilitador talentoso é conhecedor do tema e capaz de trabalhar com as falhas de desempenho reveladas na simulação para gerar discussões sobre como melhorar ou manter o desempenho clínico ou de trabalho em equipa noutros ambientes ou no futuro.

Desempenho Elemento 6

Ajuda a fechar falhas de desempenho através da discussão e ensino.

Uma vez entendida a base da falha de desempenho, é o momento de ajudar os participantes a entender como ter um melhor desempenho na próxima vez. A abordagem para minimizar as falhas de desempenho pode ser feita discutindo mudanças nas perspectivas e ações (geralmente resultando em *scores* maiores de DASH para o facilitador), ou somente mudanças nas ações.

Comportamentos positivos e eficazes incluem:

- Discutir o caso atual, procedimento, ou evento para promover o desenvolvimento de estratégias que melhorem o futuro desempenho dos participantes.
- Se o participante não desenvolve novas práticas através da discussão, usar uma aula muito curta para partilhar conhecimento, experiências ou achados de pesquisa para informar os participantes de como melhorar desempenho.
- Fornecer informações específicas ou conhecimentos cuja ausência contribuiu para a falha de desempenho.
- Usar informação que um participante partilhou para ajudar a formar novas maneiras de pensar sobre ou resolver um problema clínico ou de trabalho em equipa.
- Reforçar que a principal preocupação é o bem-estar dos pacientes.

Comportamentos negativos e ineficazes incluem:

- Não sugerir ou descobrir meios para melhorar o desempenho na prática futura.
- Dar instruções sem explicação.
- Sarcasticamente dizer aos participantes que deveriam considerar outra carreira.
- Ignorar um desempenho bom ou excelente.

Demonstra firme compreensão sobre o assunto.

Facilitadores eficientes têm experiência sobre o assunto. Tópicos no *debriefing* podem incluir muitos assuntos relacionados com a situação clínica, comportamento, trabalho em equipa, ética, etc. O facilitador discute os tópicos de forma bem informada sem exceder os limites de sua competência.

Comportamentos positivos e eficazes incluem:

- Transmitir conhecimento relevante e atual.
- Encorajar práticas corretas e de forma bem informada agir segundo princípios fundamentais.
- Estar confortável com o material, discutir e garantir que o participante compreende.
- Informar os participantes quando o limite do conhecimento do facilitador foi alcançado.
- Estar interessado em extrair o melhor conhecimento ao invés de manter aparência de omnisciência.

Comportamentos negativos e ineficazes incluem:

- Fornecer informações desatualizadas ou incorretas.
- Permitir que práticas inaceitáveis não sejam abordadas.
- Não estar aberto a aprender com outros, incluindo participantes.

[Nota: Descuidos sérios ou informações notoriamente erradas transmitidas pelo facilitador são causa de avaliar o Elemento inteiro como 1 mesmo que outras dimensões do Elemento 6 tenham sido feitas corretamente].

Atende os principais objetivos da sessão.

O avaliador deverá saber os objetivos para avaliar esse requisito.

Eventos simulados, procedimentos ou outras atividades que compõe o curriculum têm objetivos educacionais específicos. O facilitador atende os objetivos educacionais. Se outros temas são discutidos além dos objetivos pretendidos, o facilitador aparenta fazer escolhas racionais.

Comportamentos positivos e eficazes incluem:

- Discutir todos os objetivos educacionais importantes do cenário.
- Desviar o foco do curriculum quando há alto benefício educacional ou voltar a blocos fundamentais para construir objetivos importantes.
- Discutir os objetivos de maneira a demonstrar conforto e experiência em relação aos objetivos.
- Garantir que os participantes entenderam o princípio, técnica ou abordagem discutida.
- Se os objetivos não forem cumpridos, falar sobre o porquê de não ter acontecido.

Comportamentos negativos e ineficazes incluem:

- Permitir que a conversa desvie para tópicos não essenciais ao curriculum.
- Permitir que pontos chave sejam perdidos.
