# Supplementary material for: Translation of the Debriefing Assessment for Simulation in Healthcare in Portuguese and cross-cultural adaptation for Portugal and Brazil
Source: Adv Simul (Lond). 2021 Jul 7;6:25. doi: 10.1186/s41077-021-00175-z (PMC8265112; doi:10.1186/s41077-021-00175-z)
Supplement: Supplementary file 5 — Additional file 5. Different translations of DASH rater, student and instructor version. [file 41077_2021_175_MOESM5_ESM.pdf]

DASH Rater Version

T1 – Brazilian Portuguese

DASH - *Debriefing Assessment for Simulation in Healthcare* – Avaliação de *Debriefing* em Simulação Médica – Classificação ©

Instruções: Classifique a qualidade do *debriefing* utilizando escalas de efetividade com seis Elementos. O Elemento 1 classifica a introdução ao curso de simulação e não será classificada se não observou a introdução. Os Elementos abrangem Dimensões e Comportamentos pertinentes para o *debriefing* como definido no “DASH Rater’s Handbook”. Em cada Elemento, o *debriefing* pode variar de Excelente a Prejudicial. Repare que a classificação geral dos Elementos **não** é a média das classificações para cada Dimensão ou Comportamento. Pense de formal global e não matemática quando considera o impacto das Dimensões, que podem não ter o mesmo peso. O avaliador deve ponderar as Dimensões da forma mais adequada, baseado na **visão global do Elemento**. Se não for possível avaliar uma Dimensão (ex: como se lidou com um participante aborrecido, se ninguém ficou aborrecido), ignore e não permita que isso influencie a sua avaliação.

Escala de classificação

Classificação	1	2	3	4	5	6	7
Descrição	Extremamente inefetivo/Prejudicial	Consistentemente inefetivo/Muito medíocre	Bastante inefetivo/Medíocre	Um pouco inefetivo/Médio	Bastante efetivo/Bom	Consistentemente efetivo/Muito bom	Extremamente efetivo/Excelente

T2 – Portuguese

DASH - *Debriefing Assessment for Simulation in Healthcare* – Avaliação de *Debriefing* em Simulação Clínica – Classificação ©

Instruções: Classifique a qualidade do *debriefing* utilizando escalas de eficácia com seis Elementos. O Elemento 1 classifica a introdução ao exercício de simulação e não será classificada se não observou a introdução. Os Elementos abrangem Dimensões e Comportamentos pertinentes para o *debriefing* como definido no “Manual do Avaliador do DASH”. Em cada Elemento, o *debriefing* pode variar de Excelente a Prejudicial. Repare que a classificação geral dos Elementos **não** é a média das classificações para cada Dimensão ou Comportamento. Pense de formal global e não matemática quando considera o impacto das Dimensões, que podem não ter o mesmo peso. O avaliador deve ponderar as Dimensões da forma mais adequada, baseado na **visão global do Elemento**. Se não for possível avaliar uma Dimensão (ex: como se lidou com um participante descontente, se ninguém ficou descontente), ignore e não permita que isso influencie a sua avaliação.

Escala de Pontuação

Escore	**1**	**2**	**3**	**4**	**5**	**6**	**7**
Descrição	**Extremamente Ineficaz/ Prejudicial**	Consistentemente Ineficaz/ Mau Na maior parte do tempo	Ineficaz/ Medíocre	Eficaz /Médio	Bastant eficaz/ Bom	Consistentemente eficaz/ Muito Bom	**Extremamente eficaz / Excelente**

Review

DASH - *Debriefing Assessment for Simulation in Healthcare* – Avaliação de *Debriefing* em Simulação Clínica – Classificação ©

Instruções: Classifique a qualidade do *debriefing* utilizando escalas de eficácia com seis Elementos. O Elemento 1 classifica a introdução ao exercício de simulação e não será classificado se não observou a introdução. Os Elementos abrangem Dimensões e Comportamentos pertinentes para o *debriefing* como definido no “Manual do Avaliador do DASH”. Em cada Elemento, o *debriefing* pode variar de Excelente a Prejudicial. Repare que a classificação geral dos Elementos **não** é a média das classificações para cada Dimensão ou Comportamento. Pense de forma global e não matemáticamente quando considera o impacto das Dimensões, que podem não ter o mesmo peso. O avaliador deve ponderar as Dimensões da forma mais adequada, baseado na **visão global do Elemento**. Se não for possível avaliar uma Dimensão (ex: como se lidou com um participante descontente, se ninguém ficou descontente), ignore e não permita que isso influencie a sua avaliação.

Escala de Pontuação

Classificação	**1**	**2**	**3**	**4**	**5**	**6**	**7**
Descrição	**Muito Ineficaz/ Muito insatisfatório**	Consistentemente ineficaz/ Consistentemente insatisfatório	Ineficaz/ Insatisfatório	Pouco eficaz/ Satisfatório	Bastante eficaz/ Bom	Consistentemente eficaz/ Muito Bom	**Extremamente eficaz/ Excepcional**

**O Elemento 1 avalia a introdução no início de um exercício de simulação.**<br>*(Este Elemento deve ser ignorado se o avaliador não observou a introdução do curso.)*	**O Elemento 1 avalia a introdução no início de um exercício de simulação.**<br>*(Este Elemento deve ser ignorado se o avaliador não observou a introdução do curso.)*	**O Elemento 1 avalia a introdução no início de um exercício de simulação.**<br>*(Este Elemento deve ser ignorado se o avaliador não observou a introdução do curso.)*
Elemento 1<br>**Classificação Elemento 1** _________<br>**Estabelece um ambiente envolvente de aprendizagem.**	Elemento 1 **Classificação Elemento 1**<br>**Estabelece um ambiente envolvente de aprendizagem.** _________	Elemento 1 **Classificação Elemento 1**<br>**Estabelece um ambiente envolvente de aprendizagem.** _________
• Esclarece os objetivos do curso, ambiente, confidencialidade, papéis e expectativas.<br>• Estabelece um "compromisso de ficção" com os participantes.<br>• Tem em atenção detalhes logísticos.<br>• Estabelece um compromisso em respeitar os participantes e em entender as suas perspetivas.	• Clarifica todas as questões relacionadas com os objetivos do exercício, ambiente, confidencialidade, papéis e expectativas.<br>• Estabelece um "contrato de ficção" com os participantes.<br>• Esclarece os detalhes logísticos.<br>• Define o compromisso de respeitar os participantes e compreender as suas perspetivas.	• Clarifica todas as questões relacionadas com os objetivos do exercício, ambiente, confidencialidade, papéis e expectativas.<br>• Estabelece um "contrato de ficção" com os participantes.<br>• Esclarece detalhes logísticos.<br>• Define o compromisso de respeitar os participantes e compreender as suas perspectivas.
**Os Elementos 2 a 6 avaliam o *debriefing*.**	**Os Elementos 2 a 6 avaliam o debriefing.**	**Os Elementos 2 a 6 avaliam o debriefing.**
Elemento 2<br>**Classificação Elemento 2** _________<br>**Mantem um ambiente envolvente para a aprendizagem.**	Elemento 2 **Classificação Elemento 2**<br>**Mantem um ambiente envolvente para a aprendizagem.** _________	Elemento 2 **Classificação Elemento 2**<br>**Mantem um ambiente envolvente para a aprendizagem.** _________
• Esclarece os objetivos, papéis e expectativas do *debriefing.*<br>• Ajuda os participantes a envolverem-se num contexto limitado de realismo.<br>• Mostra respeito pelos participantes e preocupa-se com a sua segurança psicológica.	• Esclarece os objetivos, papéis e expectativas do *debriefing.*<br>• Ajuda os participantes a envolverem-se num contexto limitado de realismo.<br>• Mostra respeito pelos participantes e preocupa-se com a sua segurança psicológica.	• Clarifica os objetivos, papéis e expectativas do *debriefing.*<br>• Ajuda os participantes a envolverem-se num contexto de realismo limitado.<br>• Mostra respeito pelos participantes e preocupa-se com a sua segurança psicológica.
Elemento 3<br>**Classificação Elemento 3** _________<br>**Estrutura o *debriefing* de forma organizada.**	Elemento 3 **Classificação Elemento 3**<br>**Estrutura o debriefing de forma organizada.** _________	Elemento 3 **Classificação Elemento 3**<br>**Estrutura o debriefing de forma organizada.** _________
• Encoraja os participantes a partilhar as suas reações e, se necessário, orienta-os para o que aconteceu no início da simulação.<br>• Orienta a análise do desempenho dos participantes durante o *debriefing*.<br>• Colabora com os participantes no resumo da aprendizagem da sessão que termina.	• Encoraja os participantes a partilhar as suas reações e, se necessário, orienta-os na sucessão de eventos do exercício.<br>• Orienta a análise do desempenho dos participantes durante o *debriefing*.<br>• Colabora com os participantes no resumo da aprendizagem da sessão que termina.	• Encoraja os participantes a partilhar as suas reações e, se necessário, orienta a sucessão de eventos do exercício.<br>• Orienta a análise do desempenho dos participantes durante o *debriefing*.<br>• Colabora com os participantes no resumo da aprendizagem da sessão que termina.
Elemento 4<br>**Classificação Elemento 4** _________<br>**Incentiva discussões profundas.**	Elemento 4 **Classificação Elemento 4**<br>**Incentiva discussões profundas.** _________	Elemento 4 **Classificação Elemento 4**<br>**Incentiva discussões profundas.** _________

• Usa exemplos concretos e resultados como base para seus comentários e discussão.<br>• Revela seu raciocínio e opinião.<br>• Facilita a discussão com técnicas de linguagem verbal e não-verbal.<br>• Usa vídeos ou dados gravados (se disponíveis).<br>• Reconhece e gere participantes aborrecidos.	• Usa exemplos e resultados concretos como base para os seus comentários e discussão.<br>• Revela o seu raciocínio e opinião.<br>• Facilita a discussão com técnicas de linguagem verbal e não-verbal.<br>• Usa vídeos ou dados gravados (se disponíveis).<br>• Reconhece e aborda adequadamente participantes insatisfeitos.	• Usa exemplos e resultados concretos como base para os seus comentários e discussão.<br>• Revela o seu raciocínio e opinião.<br>• Facilita a discussão com técnicas de linguagem verbal e não verbal.<br>• Usa vídeos ou dados gravados (se disponíveis).<br>• Reconhece e aborda adequadamente participantes insatisfeitos.
Elemento 5<br>**Classificação Elemento 5** _________<br>**Identifica e explora falhas de desempenho.**<br>• Comenta o desempenho.<br>• Explora a origem das falhas de desempenho.	Elemento 5 **Classificação Elemento 5**<br>**Identifica e explora falhas de desempenho.** _________<br>• Comenta o desempenho.<br>• Explora a origem das limitações do desempenho.	Elemento 5 **Classificação Elemento 5**<br>**Identifica e explora falhas de desempenho.** _________<br>• Comenta o desempenho.<br>• Explora a origem das falhas de desempenho.
Elemento 6<br>**Classificação Elemento 6** _________<br>**Ajuda os participantes a perceber como podem melhorar ou manter seu bom desempenho.**<br>• Ajuda no esclarecimento das falhas de desempenho através de discussão e ensino.<br>• Demonstra domínio de conhecimento sobre o tema.<br>• Aborda todos os pontos importantes.	Elemento 6 **Classificação Elemento 6**<br>**Ajuda os participantes a perceber como podem melhorar ou manter o seu desempenho.** _________<br>• Ajuda no esclarecimento das limitações do desempenho através de discussão e ensino.<br>• Demonstra domínio de conhecimento sobre o tema.<br>• Aborda todos os pontos importantes.	Elemento 6 **Classificação Elemento 6**<br>**Ajuda os participantes a perceber como podem melhorar ou manter o seu desempenho.** _________<br>• Ajuda no esclarecimento das falhas de desempenho através de discussão e ensino.<br>• Demonstra domínio de conhecimento sobre o tema.<br>• Aborda todos os pontos importantes.
Direitos Autorais. *Center for Medical Simulation*, www.harvardmedsim.org, 2011.	Direitos Autorais. *Center for Medical Simulation*, www.harvardmedsim.org, 2011.	Direitos Autorais. *Center for Medical Simulation*, www.harvardmedsim.org, 2011.

DASH Student Version

T1 – Brazilian Portuguese

DASH - *Debriefing Assessment for Simulation in Healthcare* – Avaliação de *Debriefing* em Simulação Médica – Versão do Formando ©

Instruções: Pede-se que resuma a sua impressão sobre a introdução e *debriefing* deste exercício de simulação. Utilize a seguinte escala para classificar cada um dos seis “Elementos”. Cada Elemento é composto por diferentes Comportamentos do instrutor, descritos abaixo. Se um dos Comportamentos listados for impossível de avaliar (ex: como o instrutor lidou com pessoas aborrecidas, se ninguém esteve aborrecido), deixe o espaço em branco e não permita que isto influencie a sua avaliação. O instrutor pode ter sido bem-sucedido ou menos bem-sucedido dentro de cada Elemento. A classificação de Elemento representa a sua impressão **geral** do desempenho do instrutor no Elemento em questão. Faça o possível para classificar a **efetividade geral de todo o Elemento**, guiado pela observação dos comportamentos únicos que o definem.

Escala de classificação

Classificação	1	2	3	4	5	6	7
Descrição	Extremamente inefetivo/Prejudicial	Consistentemente inefetivo/Muito medíocre	Bastante inefetivo/Medíocre	Um pouco inefetivo/Médio	Bastante efetivo/Bom	Consistentemente efetivo/Muito bom	Extremamente efetivo/Excelente

O Elemento 1 avalia a introdução no início de um exercício de simulação.

Ignore este elemento se não tiver participado na introdução.
Se não existiu introdução e sentiu que deveria ter existido para orientá-lo, a sua avaliação deverá refletir isso.

Elemento 1

Classificação Elemento 1 _________

O instrutor preparou o cenário para uma experiência envolvente de aprendizagem.

- O instrutor apresentou-se, descreveu o ambiente do cenário, o que é esperado durante a atividade e os objetivos da aprendizagem.
- O instrutor expôs os pontos fortes e fracos da simulação e o que se pode fazer para aproveitar ao máximo a experiência em simulação médica.

T2 – Portuguese

DASH - *Debriefing Assessment for Simulation in Healthcare* – Avaliação de Debriefing em Simulação Clínica – Versão do Participante ©

Instruções: Pede-se que resuma a sua avaliação sobre a introdução e *debriefing* deste exercício de simulação. Utilize a seguinte escala para classificar cada um dos seis “Elementos”. Cada Elemento é composto por diferentes Comportamentos do instrutor, descritos abaixo. Se um dos Comportamentos listados for impossível de avaliar (ex: como o instrutor lidou com pessoas descontentes, se ninguém esteve descontente), deixe o espaço em branco e não permita que isto influencie a sua avaliação. O instrutor pode ter sido mais ou menos bem-sucedido dentro de cada Elemento. A classificação de Elemento representa a sua impressão **geral** do desempenho do instrutor no Elemento em questão. Faça o possível para classificar a **efetividade geral de todo o Elemento**, guiado pela observação dos comportamentos únicos que o definem.
.

Escala de Pontuação

Escore	**1**	**2**	**3**	**4**	**5**	**6**	**7**
Descrição	**Extremamente Ineficaz/Prejudicial**	Consistentemente Ineficaz/Muto Mau Na maior parte do tempo	Ineficaz/Medíocre	Eficaz/Médio	Muito eficaz/Bom	Consistentemente Eficaz/Muito Bom	**Extremamente Eficaz/Excelente**

O Elemento 1 avalia a introdução no início do exercício de simulação.

Ignore este elemento se não tiver participado na introdução.
Se não existiu introdução e sentiu que deveria ter existido para orientá-lo, a sua avaliação deverá refletir isso.

Elemento 1

Classificação Elemento 1 _________

O instrutor preparou o cenário para uma experiência envolvente de aprendizagem.

- O instrutor apresentou-se, descreveu a envolvência do cenário, o esperado durante a atividade e os objetivos da aprendizagem.
- O instrutor expôs os pontos fortes e as limitações da simulação e o que se pode fazer para aproveitar ao máximo a experiência em simulação clínica.

Review

DASH - *Debriefing Assessment for Simulation in Healthcare* – Avaliação de Debriefing em Simulação Clínica – Versão do Participante ©

Instruções: Pede-se que resuma a sua avaliação sobre a introdução e *debriefing* deste exercício de simulação. Utilize a seguinte escala para classificar cada um dos seis “Elementos”. Cada Elemento é composto por diferentes Comportamentos do facilitador, descritos abaixo. Se um dos Comportamentos listados for impossível de avaliar (ex: como o facilitador lidou com pessoas descontentes, se ninguém esteve descontente), deixe o espaço em branco e não permita que isso influencie a sua avaliação. O facilitador pode ter sido mais ou menos bem-sucedido dentro de cada Elemento. A classificação do Elemento representa a sua impressão **geral** do desempenho do facilitador no Elemento em questão. Faça o possível para classificar a **eficácia geral de todo o Elemento**, guiado pela observação dos comportamentos únicos que o definem.

Escala de Pontuação

Classificação	**1**	**2**	**3**	**4**	**5**	**6**	**7**
Descrição	**Muito Ineficaz/ Muito insatisfatório**	Consistentemente ineficaz/Consistentemente insatisfatório	Ineficaz/Insatisfatório	Pouco eficaz/Satisfatório	Bastante eficaz/Bom	Consistentemente eficaz/Muito Bom	**Extremamente eficaz/Excepcional**

O Elemento 1 avalia a introdução no início do exercício de simulação.

Ignore este elemento se não tiver participado na introdução.
Se não existiu introdução e sentiu que deveria ter existido para orientá-lo, a sua avaliação deverá refletir isso.

Elemento 1

Classificação Elemento 1 _______

O facilitador preparou o cenário para uma experiência envolvente de aprendizagem.

- O facilitador apresentou-se, descreveu o local do cenário, o esperado durante a atividade e os objetivos de aprendizagem.
- O facilitador expôs os pontos fortes e as limitações da simulação e o que se pode fazer para aproveitar ao máximo a experiência em simulação.

• O instrutor teve em atenção detalhes logísticos necessários, como localização da casa-de-banho, disponibilidade de alimentação e programa.<br>• O instrutor estimulou-o a partilhar os meus pensamentos e questões sobre a simulação que iria acontecer e o *debriefing* e reforçou que não iria ser envergonhado ou humilhado durante o processo.<br><br>Os Elementos 2 a 6 avaliam o *debriefing*.<br><br>Elemento 2<br>**Classificação Elemento 2 _________**<br>**O instrutor manteve um ambiente envolvente para a aprendizagem.**<br><br>• O instrutor esclareceu o objetivo do *debriefing*, o que era esperado de si e qual o papel do instrutor no *debriefing*.<br>• O instrutor reconheceu os problemas relacionados com o realismo e ajudou-o a aprender apesar de se tratar de casos simulados.<br>• Sentiu que o instrutor respeitou os participantes.<br>• O objetivo principal centrou-se na aprendizagem e não em fazer os participantes sentirem-se mal por cometer erros.<br>• Os participantes puderam partilhar pensamentos e emoções sem medo de se sentirem envergonhados ou humilhados.<br><br>Elemento 3<br>**Classificação Elemento 3 _________**<br>**O instrutor estruturou o *debriefing* de forma organizada.**<br><br>• A conversa progrediu de forma lógica em vez de saltar de ponto em ponto.<br>• No início do *debriefing,* foi encorajado a partilhar as suas reações genuínas relativamente ao caso e o instrutor considerou-as de forma séria.<br>• Durante o *debriefing*, o instrutor ajudou-o a analisar as ações e pensamentos enquanto se revia o caso.<br>• No final, foi feito um resumo do caso onde o instrutor ajudou a juntar todas as observações e relacionou o caso com diferentes formas para que possa melhorar a sua prática clínica futura.<br><br>Elemento 4<br>**Classificação Elemento 4 _________**<br>**O instrutor provocou discussões profundas que o levaram a refletir sobre o seu desempenho.**<br><br>• O instrutor usou exemplos concretos – não apenas comentários abstratos e generalizados – para o fazer pensar sobre o seu desempenho.<br>• O ponto de vista do instrutor foi claro; não teve que adivinhar o que o instrutor estaria a pensar.	• O instrutor informou detalhes logísticos necessários, como localização dos recursos sanitários, disponibilidade de alimentação e programa.<br>• O instrutor estimulou-o a partilhar os seus pensamentos e questões sobre o caso simulado e o *debriefing* e reforçou que não iriam ser constrangidos ou inferiorizados durante o processo.<br><br>Os Elementos 2 a 6 avaliam o debriefing.<br><br>Elemento 2<br>**Classificação Elemento 2 _________**<br>**O instrutor manteve um ambiente envolvente para a aprendizagem.**<br><br>• O instrutor esclareceu os objetivos do *debriefing*, o que era esperado dos participantes e qual o seu papel no *debriefing*.<br>• O instrutor reconheceu os problemas relacionados com o realismo e ajudou-o a aprender apesar de se tratar de casos simulados.<br>• Sentiu que o instrutor respeitou os participantes.<br>• O objetivo principal centrou-se na aprendizagem e não em fazer os participantes sentirem-se mal por cometer erros.<br>• Os participantes puderam partilhar pensamentos e emoções sem medo de se sentirem constrangidos ou inferiorizados.<br><br>Elemento 3<br>**Classificação Elemento 3 _________**<br>**O instrutor estruturou o *debriefing* de forma organizada.**<br><br>• A conversa progrediu de forma lógica em vez de saltar de ponto em ponto.<br>• No início do *debriefing,* foi encorajado a partilhar as suas reações genuínas relativamente ao caso e o instrutor considerou-as de forma séria.<br>• Durante o *debriefing*, o instrutor ajudou-o a analisar as ações e pensamentos enquanto se revia o caso.<br>• No final, foi feito um resumo do caso onde o instrutor ajudou a integrar todas as observações e relacionou o caso com diferentes envolvências para que possa melhorar a sua prática clínica futura.<br><br>Elemento 4<br>**Classificação Elemento 4 _________**<br>**O instrutor provocou discussões profundas que o levaram a refletir sobre seu desempenho.**<br><br>• O instrutor usou exemplos concretos – não apenas comentários abstratos e generalizados – para o fazer refletir sobre o seu desempenho.<br>• O ponto de vista do instrutor foi claro; não tive que adivinhar o seu pensamento.<br>• O instrutor ouviu e fez com que os participantes se sentissem ouvidos ao tentar incluir todos na discussão,	• O facilitador informou os detalhes logísticos necessários, como localização dos recursos sanitários, disponibilidade de alimentação e programa.<br>• O facilitador estimulou-o a partilhar os seus pensamentos e questões sobre o caso simulado e o *debriefing* e reforçou que não iriam existir constrangimentos durante o processo.<br><br>Os Elementos 2 a 6 avaliam o debriefing.<br><br>Elemento 2<br>**Classificação Elemento 2 ______**<br>**O facilitador manteve um ambiente envolvente para a aprendizagem.**<br><br>• O facilitador esclareceu os objetivos do *debriefing*, o que era esperado dos participantes e qual o seu papel no *debriefing*.<br>• O facilitador reconheceu os problemas relacionados com o realismo e ajudou-o a aprender apesar de se tratar de casos simulados.<br>• Sentiu que o facilitador respeitou os participantes.<br>• Os participantes puderam partilhar pensamentos e emoções sem medo de se sentirem constrangidos ou inferiorizados.<br><br>Elemento 3<br>**Classificação Elemento 3 ______**<br>**O facilitador estruturou o *debriefing* de forma organizada.**<br><br>• A conversa progrediu de forma lógica em vez de saltar de ponto em ponto.<br>• No início do *debriefing,* foi encorajado a partilhar as suas reações genuínas relativamente ao caso e o facilitador considerou-as de forma séria.<br>• Durante o *debriefing*, o facilitador ajudou-o a analisar as ações e pensamentos enquanto se revia o caso.<br>• No final, foi feito um resumo do caso onde o facilitador o ajudou a integrar todas as observações e **relacionou o caso de diferentes maneiras** para que possa melhorar a sua prática clínica futura.<br><br>Elemento 4<br>**Classificação Elemento 4 ______**<br>**O facilitador provocou discussões profundas que o levaram a refletir sobre o seu desempenho.**<br><br>• O facilitador usou exemplos concretos – não apenas comentários abstratos e generalizados – para fazê-lo refletir sobre o seu desempenho.<br>• O ponto de vista do facilitador foi claro; não teve que adivinhar o seu pensamento.<br>• O facilitador ouviu e fez com que os participantes se sentissem ouvidos ao tentar incluir todos na discussão,

• O instrutor ouviu e fez com que os participantes se sentissem ouvidos ao tentar incluir todos, interpretar os comentários e utilizar linguagem não-verbal, como contacto visual e acenar com a cabeça, etc.<br>• O instrutor usou vídeos ou dados gravados para apoiar a análise e aprendizagem.<br>• Se alguém ficou aborrecido no *debriefing*, o instrutor foi respeitador e construtivo ao tentar ajuda-lo a lidar com a situação.	interpretar os comentários e utilizar linguagem não-verbal, como olhar nos olhos dos participantes ou acenar com a cabeça.<br>• O instrutor usou vídeos ou dados gravados para apoiar a análise e aprendizagem.<br>• Se alguém ficou descontente no *debriefing*, o instrutor foi respeitador e construtivo ao tentar ajuda-lo a lidar com a situação.	interpretar os comentários e utilizar linguagem não verbal, como olhar nos olhos dos participantes ou acenar com a cabeça.<br>• O facilitador usou vídeos ou dados gravados para apoiar a análise e aprendizagem.<br>• Se alguém ficou descontente no *debriefing*, o facilitador foi respeitador e construtivo ao tentar ajuda-lo a lidar com a situação.
Elemento 5<br>**Classificação Elemento 5** _________<br>**O instrutor identificou o que fez bem e mal – e porquê.**<br><br>• Recebeu comentários concretos sobre o seu desempenho ou do grupo baseados em declarações objetivas e honestas do instrutor.<br>• O instrutor ajudou a explorar o que estava a pensar ou o que tentava alcançar nos momentos-chave.	Elemento 5<br>**Classificação Elemento 5** _________<br>**O instrutor identificou o que fizeremos melhor e pior – e porquê**<br><br>• Recebeu comentários concretos sobre o seu desempenho e/ou do grupo baseados em declarações objetivas e honestas do instrutor.<br>• O instrutor ajudou a explorar o que pensava ou o que tentava alcançar nos momentos-chave.	Elemento 5<br>**Classificação Elemento 5** _______<br>**O facilitador identificou o que fizeremos melhor e pior – e por que.**<br><br>• Recebeu comentários concretos sobre o seu desempenho e/ou do grupo baseados em declarações objetivas e honestas do facilitador.<br>• O facilitador ajudou a explorar o que pensava ou o que tentava alcançar nos momentos chave.
Elemento 6<br>**Classificação Elemento 6** _________<br>**Ajudou os participantes a perceber como podem melhorar ou manter seu bom desempenho.**<br><br>• O instrutor ajudou-o a aprender como pode melhorar áreas de fraqueza ou como repetir um bom desempenho.<br>• O instrutor era experiente e usou os seus conhecimentos para o ajudar a perceber como pode desenvolver um bom desempenho no futuro.<br>• O instrutor garantiu que todos os pontos importantes foram abordados.	Elemento 6<br>**Classificação Elemento 6** _________<br>**Ajudou os participantes a perceber como podem melhorar ou manter um bom desempenho.**<br><br>• O instrutor ajudou-o a aprender como pode melhorar deficiências ou como repetir um bom desempenho.<br>• O instrutor era experiente e usou o seu conhecimento para ajudá-lo a perceber como pode melhorar o desempenho no futuro.<br>• O instrutor garantiu que todos os pontos importantes foram abordados.	Elemento 6<br>**Classificação Elemento 6** _______<br>**Ajudou os participantes a perceber como podem melhorar ou manter um bom desempenho.**<br><br>• O facilitador ajudou-o a aprender como pode melhorar deficiências e/ou como repetir um bom desempenho.<br>• O facilitador era experiente e usou o seu conhecimento para o ajudar a perceber como pode melhorar o seu desempenho no futuro.<br>• O facilitador garantiu que todos os pontos importantes foram abordados.
Direitos Autorais. *Center for Medical Simulation*, www.harvardmedsim.org, 2011.	Direitos Autorais. *Center for Medical Simulation*, www.harvardmedsim.org, 2011.	Direitos Autorais. *Center for Medical Simulation*, www.harvardmedsim.org, 2011.

DASH Insctructor Version

T1 – Brazilian Portuguese

DASH - *Debriefing Assessment for Simulation in Healthcare* – Avaliação de *Debriefing* em Simulação Médica – Versão do Instrutor ©

Instruções: Faça uma auto-avaliação sobre o seu desempenho na introdução e no *debriefing* deste exercício de simulação. Utilize a seguinte escala para classificar cada um dos seis “Elementos”. Para cada Elemento, os Comportamentos são dado para indicar o desempenho positivo no Elemento em questão. Tente classificar a sua **efetividade global para cada Elemento** guiado pelos Comportamentos que o definem. Se um dos Comportamentos listados for impossível de avaliar (ex: como o instrutor lidou com pessoas aborrecidas, se ninguém esteve aborrecido), deixe o espaço em branco e não permita que isto influencie a sua avaliação. Pode ter sido bem-sucedido ou menos bem-sucedido dentro de cada Elemento. A classificação de Elemento representa a sua impressão **geral** do seu desempenho no Elemento em questão. O Elemento 1 avalia a apresentação no início do exercício de simulação. Os Elementos 2 a 6 avaliam o *debriefing*.

Escala de classificação

Classificação	1	2	3	4	5	6	7
Descrição	Extremamente inefetivo/ Prejudicial	Consistentemente inefetivo/Muito medíocre	Bastante inefetivo/Medíocre	Um pouco inefetivo /Médio	Bastante efetivo/Bom	Consistentemente efetivo/ Muito bom	Extremamente efetivo/Excelente

O Elemento 1 avalia a introdução no início de um exercício de simulação.
Ignore este elemento se não tiver participado na introdução.

Elemento 1
Classificação Elemento 1 ________
Preparei o cenário para uma experiência envolvente de aprendizagem.

- Apresentei-me, descrevi o ambiente do cenário, o que é esperado durante a atividade.
- Apresentei os objetivos da aprendizagem e clarifiquei questões de confidencialidade.
- Expus os pontos fortes e fracos da simulação e o que os participantes podem fazer para aproveitar ao máximo a experiência em simulação médica.

T2 – Portuguese

DASH - *Debriefing Assessment for Simulation in Healthcare* – Avaliação do *Debriefing* em Simulação Clínica – Versão do Instrutor ©

Instruções: Faça uma auto-avaliação do seu desempenho na introdução e no *debriefing* deste exercício de simulação. Utilize a seguinte escala para classificar cada um dos seis “Elementos”. Para cada Elemento, os Comportamentos são referências para indicar o desempenho positivo no Elemento em questão. Tente classificar a sua **eficácia global para cada Elemento** guiado pelos comportamentos que o caracterizam. Se um dos Comportamentos listados for impossível de avaliar (ex: como o instrutor lidou com pessoas descontentes, se ninguém esteve descontente), deixe o espaço em branco e não permita que isto influencie a sua avaliação. Você pode ter sido mais, ou menos bem-sucedido dentro de cada Elemento. A classificação de Elemento representa sua impressão **geral** de seu desempenho no Elemento em questão. A classificação de Elemento representa a sua impressão **geral** do seu desempenho no Elemento em questão. O Elemento 1 avalia a introdução no início do exercício de simulação. Os Elementos 2 a 6 avaliam o *debriefing*.

Escala de Pontuação

Classificação	**1**	**2**	**3**	**4**	**5**	**6**	**7**
Descrição	**Extremamente Ineficaz/ Muito insatisfatório**	Consistentemente ineficaz/ Consistentemente insatisfatório	Ineficaz/ Insatisfatório	Pouco eficaz/ Satisfatório	Bastante eficaz/ Bom	Consistentemente eficaz/ Muito Bom	**Extremamente eficaz/ Excelente**

O Elemento 1 avalia a introdução prévia ao exercício de simulação.
Ignore este elemento se não tiver participado na introdução.

Elemento 1
Classificação
Elemento 1
Preparei o cenário para uma experiência de aprendizagem envolvente
____

- Apresentei-me, descrevi o local do cenárioe o esperado durante a atividade.
- Apresentei os objetivos da aprendizagem e clarifiquei as questões de confidencialidade.
- Expus os pontos fortes e as limitações da simulação e o que os participantes podem fazer para aproveitar ao máximo a experiência em simulação clínica.

Review

DASH - *Debriefing Assessment for Simulation in Healthcare* – Avaliação do *Debriefing* em Simulação Clínica – Versão do Facilitador ©

Instruções: Faça uma autoavaliação do seu desempenho na introdução e no *debriefing* deste exercício de simulação. Utilize a seguinte escala para classificar cada um dos seis “Elementos”. Para cada Elemento, os Comportamentos são referências para indicar o desempenho positivo no Elemento em questão. Tente classificar a sua **eficácia global para cada Elemento** guiado pelos comportamentos que o caracterizam. Se um dos Comportamentos listados for impossível de avaliar (ex: como o facilitador lidou com pessoas descontentes, se ninguém esteve descontente), deixe o espaço em branco e não permita que isso influencie a sua avaliação. Pode ter sido mais, ou menos bem-sucedido dentro de cada Elemento. A classificação do Elemento representa a impressão **geral** do seu desempenho no Elemento em questão. O Elemento 1 avalia a introdução no início do exercício de simulação. Os Elementos 2 a 6 avaliam o *debriefing*.

Escala de Pontuação

Classificação	**1**	**2**	**3**	**4**	**5**	**6**	**7**
Descrição	**Muito Ineficaz/ Muito insatisfatório**	Consistentemente ineficaz/ Consistentemente insatisfatório	Ineficaz/ Insatisfatório	Pouco eficaz/ Satisfatório	Bastante eficaz/ Bom	Consistentemente eficaz/ Muito Bom	**Extremamente eficaz/ Excepcional**

O Elemento 1 avalia a introdução prévia ao exercício de simulação.
Ignore este elemento se não tiver participado na introdução.

Elemento 1
Classificação
Elemento 1
Preparei o cenário para uma experiência de aprendizagem envolvente
____

- Apresentei-me, descrevi o local do cenário e o esperado durante a atividade.
- Apresentei os objetivos da aprendizagem e clarifiquei questões de confidencialidade.
- Expus os pontos fortes e as limitações da simulação e o que os participantes podem fazer para aproveitar ao máximo a experiência em simulação clínica.

• Tive em atenção detalhes logísticos necessários, como localização da casa-de-banho, disponibilidade de alimentação e programa.<br>• Estimulei os participantes a partilhar os seus pensamentos e questões sobre a simulação que iria acontecer e o *debriefing* e reforcei que não iriam ser envergonhados ou humilhados durante o processo.<br><br>Os Elementos 2 a 6 avaliam o *debriefing*.<br><br>Elemento 2<br>**Classificação Elemento 2** _________<br>**Mantive um ambiente envolvente para a aprendizagem.**<br><br>• Esclareci o objetivo do *debriefing*, o que era esperado dos participantes e qual o meu papel no *debriefing*.<br>• Reconheci os problemas relacionados com o realismo e ajudei os participantes a aprender apesar de se tratar de casos simulados.<br>• Mostrei respeito pelos participantes.<br>• Assegurei-me que o objetivo principal se centrou na aprendizagem e não em fazer os participantes se sentirem mal por cometer erros.<br>• Estimulei os participantes a partilharem pensamentos e emoções sem medo de se sentirem envergonhados ou humilhados.<br><br>Elemento 3<br>**Classificação Elemento 3** _________<br>**Estruturei o *debriefing* de forma organizada.**<br><br>• Conduzi a conversa a progredir de forma lógica em vez de saltar de ponto em ponto.<br>• No início do *debriefing,* encorajei os participantes a partilharem as suas reações genuínas relativamente ao caso e considerei-as de forma séria.<br>• Durante o *debriefing*, ajudei os participantes a analisar as suas ações e pensamentos enquanto se revia o caso.<br>• No final, foi feito um resumo do caso onde ajudei os participantes a juntar todas as observações e relacionei o caso com diferentes formas para que eles possam melhorar a sua prática clínica futura.<br><br>Elemento 4<br>**Classificação Elemento 4** _________<br>**Provoquei discussões profundas que levaram os participantes a refletir sobre o seu desempenho.**	• Informei detalhes logísticos necessários, como localização dos recursos sanitários, disponibilidade de alimentação e programa.<br>• Estimulei os participantes a compartilhar os seus pensamentos e questões sobre o caso simulado e o *debriefing,* e reforcei que não iriam ser constrangidos ou inferiorizados durante o processo.<br><br>Os Elementos 2 a 6 avaliam o *debriefing*<br><br>Elemento 2<br>**Classificação**<br>**Elemento 2**<br>**Mantive um ambiente envolvente para a aprendizagem**<br>_________<br><br>• Esclareci os objetivos do *debriefing*, o que era esperado dos participantes e qual o meu papel no *debriefing*.<br>• Reconheci os problemas relacionados com o realismo e ajudei os participantes a aprender apesar de se tratar de casos simulados.<br>• Mostrei respeito pelos participantes.<br>• Assegurei-me que o objetivo principal se centrou na aprendizagem e não em fazer os participantes sentirem-se mal por cometer erros.<br>• Estimulei os participantes a partilhar pensamentos e emoções sem medo de se sentirem constrangidos ou inferiorizados.<br><br>Elemento 3<br>**Classificação Elemento 3**<br>**Estruturei o *debriefing* de forma organizada.**<br>_________<br><br>• Conduzi a conversa progredindo de forma lógica, em vez de saltar de ponto em ponto.<br>• No início do *debriefing,* encorajei os participantes a partilharem as suas reações genuínas relativamente ao caso e considerei-as de forma séria.<br>• Durante o *debriefing*, ajudei os participantes a analisar as suas ações e pensamentos enquanto se revia o caso.<br>• No final, foi feito um resumo do caso onde ajudei os participantes a integrar todas as observações e relacionei o caso de diferentes maneiras para que eles possam melhorar a sua prática clínica futura.<br><br>Elemento 4<br>**Classificação**<br>**Elemento 4**<br>**Provoquei discussões profundas que levaram os participantes a refletir sobre o seu desempenho.**<br>_________	• Informei detalhes logísticos necessários, como localização dos recursos sanitários, disponibilidade de alimentação e programa.<br>• Estimulei os participantes a compartilhar pensamentos e questões sobre o caso simulado e o *debriefing,* e reforcei que não iriam ser constrangidos ou inferiorizados durante o processo.<br><br>Os Elementos 2 a 6 avaliam o *debriefing*<br><br>Elemento 2<br>**Classificação**<br>**Elemento 2**<br>**Mantive um ambiente envolvente para a aprendizagem**<br>_________<br><br>• Esclareci os objetivos do *debriefing*, o que era esperado dos participantes e qual o meu papel no *debriefing*.<br>• Reconheci os problemas relacionados com o realismo e ajudei os participantes a aprender apesar de se tratarem de casos simulados.<br>• Mostrei respeito pelos participantes.<br>• Assegurei que o objetivo principal se centrou na aprendizagem e não em fazer com que os participantes se sentissem mal por cometerem erros.<br>• Estimulei os participantes a partilharem pensamentos e emoções sem medo de se sentirem constrangidos ou inferiorizados.<br><br>Elemento 3<br>**Classificação Elemento 3**<br>**Estruturei o *debriefing* de forma organizada.**<br>_________<br><br>• Conduzi a conversa de forma lógica, em vez de saltar de ponto em ponto.<br>• No início do *debriefing,* incentivei os participantes a partilharem as suas reações genuínas relativamente ao caso e considerei-as de forma séria.<br>• Durante o *debriefing*, ajudei os participantes a analisar as suas ações e pensamentos enquanto revia o caso.<br>• No final, foi feito um resumo do caso onde ajudei os participantes a integrar todas as observações e abordei o caso de diferentes maneiras para que eles possam melhorar a sua prática clínica futura.<br><br>Elemento 4<br>**Classificação**<br>**Elemento 4**<br>**Promovi discussões que levaram os participantes a refletir sobre o seu desempenho.**<br>_________

• Utilizei exemplos concretos – não apenas comentários abstratos e generalizados – para que os participantes pudessem pensar sobre o seu desempenho.<br>• O meu ponto de vista foi claro; não forcei os participantes a terem que adivinhar o que eu estaria a pensar.<br>• Ouvi e fiz com que os participantes se sentissem ouvidos ao tentar incluir todos, interpretar os comentários e utilizar linguagem não-verbal, como contacto visual e acenar com a cabeça, etc.<br>• Utilizei vídeos ou dados gravados para apoiar a análise e aprendizagem (Quando disponível).<br>• Se alguém ficou aborrecido no *debriefing*, fui respeitador e construtivo ao tentar ajuda-lo a lidar com a situação.<br><br>Elemento 5<br>**Classificação Elemento 5** _________<br>**Identifiquei o que fizeram bem e mal – e porquê.**<br><br>• Realizei comentários concretos sobre o desempenho dos participantes ou do grupo baseados em declarações objetivas e no meu ponto de vista honesto.<br>• Ajudei os participantes a explorar o que estavam a pensar ou o que tentavam alcançar nos momentos-chave.<br><br>Elemento 6<br>**Classificação Elemento 6** _________<br>**Ajudei os participantes a perceber como podem melhorar ou manter seu bom desempenho.**<br><br>• Ajudei os participantes a aprender como podem melhorar áreas de fraqueza ou como repetir um bom desempenho.<br>• Usei os meus conhecimentos e experiências para ajudar os participantes a perceber como podem desenvolver um bom desempenho no futuro.<br>• Garanti que todos os pontos importantes foram abordados.<br>•<br>Direitos Autorais. *Center for Medical Simulation*, www.harvardmedsim.org, 2011.	• Utilizei exemplos concretos – não apenas comentários abstratos e generalizados – para que os participantes pudessem refletir sobre o seu desempenho.<br>• O meu ponto de vista foi claro; não forcei os participantes a adivinhar o meu pensamento.<br>• Ouvi e fiz com que os participantes se sentissem ouvidos ao incluir todos da discussão, interpretar os comentários e utilizar linguagem não-verbal, como olhar nos olhos dos participantes ou acenar com a cabeça.<br>• Utilizei vídeos ou dados gravados para apoiar a análise e aprendizagem (quando disponível).<br>• Se alguém ficou descontente com o *debriefing*, fui respeitador e construtivo ao tentar ajudá-lo a lidar com a situação.<br><br>Elemento 5<br>**Classificação Elemento 5**<br>**Identifiquei o que fizeram melhor e pior – e porquê.**<br>__________<br><br>• Realizei comentários concretos sobre o desempenho dos participantes e/ou do grupo baseados em declarações objetivas e no meu ponto de vista honesto.<br>• Ajudei os participantes a explorar o que pensavam ou o que tentavam alcançar nos momentos-chave.<br><br>Elemento 6<br>**Classificação Elemento 6**<br>**Ajudei os participantes a perceber como podem melhorar ou manter um bom desempenho.**<br>__________<br><br>• Ajudei os participantes a aprender como podem melhorar deficiências ou como repetir um bom desempenho.<br>• Usei o meu conhecimento e experiência para ajudar os participantes a perceber como podem melhorar o desempenho no futuro.<br>• Garanti que todos os pontos importantes foram abordados.<br><br>Direitos Autorais. *Center for Medical Simulation*, www.harvardmedsim.org, 2011.	• Utilizei exemplos concretos – não apenas comentários abstratos e generalizados – para que os participantes pudessem refletir sobre o seu desempenho.<br>• O meu ponto de vista foi claro; não forcei os participantes a adivinhar o meu pensamento.<br>• Ouvi e fiz com que os participantes se sentissem ouvidos ao incluir todos na discussão, interpretar os comentários e utilizar linguagem não verbal, como olhar nos olhos dos participantes ou acenar com a cabeça.<br>• Utilizei vídeos ou dados gravados para apoiar a análise e aprendizagem (quando disponível).<br>• Se alguém ficou descontente com o *debriefing*, fui respeitador e construtivo ao tentar ajudá-lo a lidar com a situação.<br><br>Elemento 5<br>**Classificação Elemento 5**<br>**Identifiquei o que fizeram melhor e pior – e por que.**<br>__________<br><br>• Realizei comentários concretos sobre o desempenho dos participantes e/ou do grupo baseados em declarações objetivas e no meu ponto de vista.<br>• Ajudei os participantes a explorar o que pensavam ou o que tentavam alcançar nos momentos chave.<br><br>Elemento 6<br>**Classificação Elemento 6**<br>**Ajudei os participantes a perceber como podem melhorar ou manter um bom desempenho.**<br>__________<br><br>• Ajudei os participantes a aprenderem como podem melhorar deficiências ou como repetir um bom desempenho.<br>• Usei o meu conhecimento e experiência para ajudar os participantes a perceber como podem melhorar o seu desempenho no futuro.<br>• Garanti que todos os pontos importantes foram abordados.<br><br>Direitos Autorais. *Center for Medical Simulation*, www.harvardmedsim.org, 2011.
