# Supplementary material for: Translation of the Debriefing Assessment for Simulation in Healthcare in Portuguese and cross-cultural adaptation for Portugal and Brazil
Source: Adv Simul (Lond). 2021 Jul 7;6:25. doi: 10.1186/s41077-021-00175-z (PMC8265112; doi:10.1186/s41077-021-00175-z)
Supplement: Supplementary file 6 — Additional file 6. Back translation of elements 2 and 5. [file 41077_2021_175_MOESM6_ESM.pdf]

DASH Manual – Elements 2 and 5

Elemento 2

MANTÉM UM AMBIENTE DE APRENDIZAGEM ENVOLVENTE.
Este elemento sobrepõe-se claramente ao Elemento 1 e engloba a capacidade do facilitador para manter um bom ambiente de aprendizagem. No início do debriefing, especialmente no primeiro do dia, e ao longo do debriefing, o instrutor deve ajudar a esclarecer aos participantes sobre o que é esperado deles. O facilitador deve garantir que os alunos se sintam confiantes em relação à segurança do ambiente para partilhar os seus pensamentos e sentimentos – isto é, eles não devem sentir-se envergonhados ou humilhados – e que o foco é no aprendizado e não em "apanhar" o erro de alguém.

Elemento 2 – Dimensões

Esclarece os objetivos, papéis e expectativas do debriefing
O debriefing flui melhor e os participantes ficam mais envolvidos quando entendem o que eles estão a fazer, o seu papel e o papel do instrutor e o que é esperado deles.

Comportamentos positivos e eficazes incluem:

- Esclarece os tópicos que serão abordados (por exemplo, "Eu gostaria de discutir como identificar arritmias.")
- Esclarece o papel dos participantes durante o debriefing.
- Solicita que os participantes participem das discussões e tentem fazer uma autorreflexão.
- Descreve o papel do facilitador explicitamente: facilitar a discussão, comentar o desempenho observado na simulação e atuar como um especialista em sua área de experiência (por exemplo, PALS, ACLS, CRM, trabalho em equipa, Clínica); para assegurar que os objetivos do treino sejam alcançados.

Comportamentos negativos e ineficazes incluem:

- Não falar sobre os papéis do facilitador ou dos participantes.
- Deixar vagos ou não declarar os objetivos do debriefing.
- Não dar tempo para perguntas ou comentários dos alunos.
- Não se ajustar às expressões verbais e não verbais feitas pelos participantes que indicam que eles estão confusos com os objetivos.

Ajudar os participantes a envolverem-se num contexto de realismo limitado
Frequentemente os participantes não apreciam (ou ressentem-se) as limitações do realismo das simulações até passarem por uma simulação específica. Eles querem ter um bom desempenho e sentem-se mal quando isso não acontece. Se os participantes se sentirem enganados ou frustrados pela tecnologia de simulação ou se preocuparem em não serem vistos de forma competente, vão reclamar. Às vezes ficam simplesmente frustrados quando a limitação do realismo confunde o processo de resolução do problema. Os facilitadores podem ajudar os alunos a abordar essas questões, reconhecendo a legitimidade de suas queixas, mas mantendo o debriefing focado nos objetivos de aprendizagem e não no equipamento. Os instrutores reforçam a seriedade e o realismo do cenário ou procedimentos simulados, tratando-os como casos reais e tratando os manequins, pacientes simulados/atores profissionais como se fossem pessoas com integridade e motivações reais. Por exemplo, se o ator está simulando um médico que está agressivo, a gritar, o facilitador deve ajudar os participantes a explorar quais são os reais problemas daquele personagem.

Comportamentos bons e efetivos incluem:

- Reforçar com os participantes que as partes físicas do simulador são diferentes da vida real.
- Reforçar que os participantes podem agir de forma um pouco diferente num ambiente simulado.

Element 2

MAINTAIN AN ENGAGING LEARNING ENVIROMENT
This element clearly overlaps element 1 and has to do with how to maintain a good learning environment. In the beginning of the debriefing, especially in the first day, and during the debriefing, the instructor can help participants be clear about what is expected of them. Facilitator must guarantee that students feel that the environment is safe for sharing thoughts and feelings. That is, they must not be shamed or humiliated – and that the focus is on learning and not on "catching" people in a mistake.

Element 2 – Dimensions

To clarity debriefing objectives, roles, and expectations
Debriefing flows better and participants engage more when they understand what they are doing, as well as their role and instructors' role, and what is expected of them.

Positive and efficient behavior include:
Clarify topics that will be approached (for example, "I would like to discuss how to find out arrhythmias causes".)
Clarify role of participants throughout the debriefing.
Request participants to take part on discussions and attempt to do self-reflection.
Explicitly state role of facilitator: to facilitate discussion, comment on performance observed in simulation and act as specialist in his/her area of experience (for example, PALS, ACLS, CRM, team working, clinic); to assure that goals of training are achieved.

Negative behaviors and inefficiency include:

- Leaving facilitators or participants roles undefined.
- Being vague or not define debriefing objectives.
- Not providing time for students to make questions or comments.
- Not justifying verbal and non-verbal expressions made by participants indicating that they are confused with objectives.

Help participants engage in a limited-realism context
Participants often do not notice (or feel) limited realisms of simulations until they experience a specific simulation. They require a good performance and feel uncomfortable when what they want do not occur. If participants feel tricked or frustrated by the simulation technology or concerned they will not be seen as competent, they will complaint. Sometimes they are simply frustrated when limited realism is confused with a problem of solving process. Facilitators can help students to approach these issues by acknowledging the legitimacy of their complaints, but keeping the debriefing focused on the learning objective and not on simulation technology. Instructors reinforce the scenario seriousness and realism or simulated procedures, and treat them as real cases and also treat mannequins, simulated patients/professional actors as they were people with integrity and real motivations. For example, if the actor is playing as a physician that is angry and yelling, the facilitator must help participants to explore what are the real problems of the character.

Good and effective behaviors include:

- Reinforcing with participants that physical parts of simulator are different in real life.
- Reinforcing that participants can act in little different way in simulated environment.
- Reinforcing or showing that despite limitations, simulation is

- Reforçar ou demonstrar que apesar das limitações da simulação, ainda assim é um recurso útil para discussão (por exemplo: comparando-se os eventos simulados com situações da vida real).
- Reconhecer os problemas ou reclamações dos participantes parafraseando e aceitando essas queixas. (Por exemplo: "Está frustrado com os sons respiratórios. Sim, eles não são ótimos.").
- Após validar a experiência dos participantes, direcionar a discussão para pontos produtivos (por exemplo: "É verdade, pode ser difícil discernir os sons respiratórios num manequim e eu compreendo isso". Eu gostaria de saber se alguém, alguma vez teve dificuldades de identificar os sons respiratórios na vida real e como isso foi para você?)
- Reconhecer que há certa estranheza em "simular".

Comportamentos negativos e ineficazes incluem:
- Discutir com o aluno o realismo do cenário;
- Negar a legitimidade das queixas dos participantes;
- Mostrar ressentimento pelos participantes não terem se envolvido completamente na simulação;
- Rir ou depreciar a tecnologia ou atores utilizados na simulação;
- Fazer com que os alunos se sintam tolos por terem integrado por inteiro no caso, procedimento ou evento apresentado.

Transmitir respeito pelos alunos e preocupar-se com a sua segurança psicológica
Transmitir respeito e realizar considerações positivas aos participantes ajuda a criar um contexto de conversa informal necessário para um bom debriefing. Os instrutores criam um ambiente favorável quando tratam os participantes como membros de uma respeitada equipe levando em consideração as suas categorias profissionais. Comunicar esse respeito pode andar de mãos dadas com críticas pontuais ao desempenho do participante. Não é necessário esconder os julgamentos, mas é necessário realizar as questões e respeitosamente ouvir as respostas.

Comportamentos bons e efetivos incluem:
- Demonstrar curiosidade genuína incentivando os participantes a partilharem os seus pensamentos e ouvirem as suas respostas;
- Realizar perguntar abertas que encorajem os participantes a explorar suas ideias;
- Realizar anotações ou lembretes de comentários realizados pelos participantes e realizar conexões ao longo do debriefing;
- Criticar os comportamentos e não as pessoas (Por exemplo, dizendo, " Gritar com a enfermeira não fez com que você tivesse as respostas que você precisava", ao invés, "Você é cabeça quente, né?")

Comportamentos negativos e ineficazes incluem:
- Perguntar e responder suas próprias perguntas;
- Falar em cima das observações dos participantes;
- Humilhar os participantes ou ridicularizar as suas respostas;
- Seguir uma linha de questões com a intenção de fazer o participante parecer ou se sentir estúpido;
- Usar sarcasmo ou ironia nas discussões sobre as ações ou pensamentos dos participantes

Elemento 5

Identifica e explora falhas de desempenho
Ao avaliar esse Elemento, considerar a capacidade do facilitador descrever a falha de desempenho – isto é, a diferença entre o desempenho atual e um desempenho ótimo. Os facilitadores deveriam dar aos participantes um parecer concreto sobre seu desempenho. Além de identificar, o facilitador deveria explorar as causas dessa falha. Quando um desempenho é sub-ótimo, o facilitador trabalha com o participante para analisar como as "estruturas" (incluindo conhecimentos a atitudes) e habilidades do participante levaram a falha de desempenho. Se um aluno vai bem, não significa que não há nada a

still a useful resource for discussion (for example: comparing the simulated events with real life situations).
- Acknowledging problems or complaints of participants by paraphrasing and accepting complaints. (For example: "You were frustrated with the breath sounds. Yes, they are not very good.").
- After validate experience of participants, directs discussion for fruitful areas (for example: "Yes, It's true that to understand breath sounds in a mannequin can be difficult, so I can understand that". I wonder if anyone had trouble diagnosis breath sounds in real life and what that´s like for you?)
- Recognizing if any discomfort to simulate exists.

Negative and inefficient behaviors include:
- Arguing with the student about realism of the scenario;
- Denying the legitimacy of participants' complaints;
- Showing resentment that participants did not fully engaged in the simulation;
- Laughing at or belittling the technology, or characters in the simulation;
- Making students feeling ridicule to be part of the case, procedure or event.

Convey respect for students and be concerned about their psychological safety
Conveying respect and positive considerations for participants to help in the creation of a context of informal conversation needed for rich debriefing. Instructors generate a favorable environment when they treat participants as respected and competent team members taking into consideration their professional categories. Communicate on this regard can be associated with specific critics for participants' performance. It does not required "sugarcoating" one's judgments, but it requires asking questions and respectfully listening to the answers.

Good and effective behaviors include:
- Showing genuine curiosity by provoking participants to share their thoughts and listen to answers;
- Making open questions that encourage participants to explore their ideas;
- Making notes or reminders of comments done by participants and promoting connections throughout the debriefing;
- Criticizing behavior and not people (for example, "Yelling with a nurse does not guarantee that one's gets the answer he/she needs", on the other hand, "You are nervous, aren't you?

Negative and inefficient behavior include:
- Asking and answering one's own questions;
- Talking over other participants' observations;
- Demeaning participants or ridiculing their answers;
- Asking and answering one's own questions;
- Following a line of questioning with intention of doing participant seem or feel stupid;
- Using sarcasm or irony to discuss participants' thoughts.

Element 5

Identify and explore performance gaps
To evaluate this Element, considering ability of facilitator to describe performance gaps – that is, the difference between the current performance and optimal performance. Facilitators should give participants a concrete feedback about their performance. In addition to identification, facilitator should explore causes of fail. When performance is suboptimal, the facilitator explores along with participant how "structures" (including knowledge and attitudes) and participants' skills led to performance fail. If a learner performance went well, this good result does not mean that there is

discutir: na situação de desempenho bom ou excelente, o instrutor auxilia o participante a identificar os conhecimentos, habilidades e atitudes que levaram a esse bom desempenho.

Dimensões Elemento 5
Fornece um parecer sobre o desempenho.
Quando estão a aprender uma competência complexa, os alunos por vezes não tem uma noção clara de como estão a progredir como uma pessoa aprendendo a guiar pode ter problemas para perceber que está na faixa. Os alunos precisam de comentários, e isso habitualmente significa realçar a distância que existe entre onde eles querem ir e onde eles estão. Um parecer claro, apreciação crítica sobre como o desempenho dos participantes está aquém, adequado ou além das expectativas para esse atividade de simulação é crucial para aprendizagem.

Comportamentos positivos e eficazes incluem:

- Expor julgamentos ou críticas ao desempenho de participantes de forma específica- e ligada a ações. (Ex. ao invés de dizer “comunicação foi ruim”, dizer “Eu não vi você olhar nos olhos da paciente ou parafrasear o que ela disse.”)
- Comentar no desempenho positivo e não só no negativo.
- Ser direto porém respeitoso. (Ex. “Eu não ouvi você se desculpar ao paciente pelo erro. Penso que isso contribuiu para ele ficar irritado”; ex. “Eu não vi você fazer dupla verificação. Eu me preocupo pois isso aumenta o risco de um erro de medicação.”)

Comportamentos negativos e ineficazes incluem:
- Falar em revelar opinião ou julgamento quando descreve observações sobre desempenho dos participantes
- Disfarçar criticismos embelezando, camuflando ou usando a “técnica do sanduíche” (afirmação positiva seguida de criticismo seguida de afirmação positiva).
- Fazer perguntas tendenciosas esperando que participantes exponham a falha de desempenho que o instrutor observou, mas não quer dizer.
- Expressar julgamento em tom sarcástico, nocivo, ofensivo ou desrespeitoso (ex. “não me admira que o paciente tenha começado a gritar! Que parte de “desculpa” é tão difícil de dizer?!”)

Explora a base da falha de desempenho
Explorar o racional ou força motriz para os participantes estarem onde eles estão ao invés de onde poderiam estar é uma característica distinta de um bom debriefing. É útil ajudar participantes a entender como suas estruturas (como conhecimentos, premissas, crenças ou sentimentos) contribuem para seu desempenho. Os facilitadores deveriam ajudar participantes a explorar essas fundações das suas ações.
Ocasionalmente é necessário por limites de tempo ou suficiente pelo objetivo do curso focar somente nas ações corretas ao invés de ajudar participantes a explorar e repensar suas <u>estruturas</u>. Um debriefing que foca exclusivamente nas ações geralmente não serão classificados acima de 4 e raramente receberão uma nota 5 nesse elemento.
Comportamentos positivos e eficazes incluem:
- Ir além do “o que” para o “porque”, isto é, explorar porque participantes tomaram as ações sem focar exclusivamente nos prós e contras de cada ação.
- Encorajar participantes a refletir no que estavam a pensar durante o cenário.
- Fornecer críticas específicas e ligadas a ações. Ex. “Eu não o ouvi resumir as preocupações da mãe”. Ou “Estava com os seus ombros encolhidos – consegue ver no vídeo?- penso que isso pode ter contribuído com a fadiga e desconforto que você sentiu durante a laparoscopia. Tente abaixar os ombros na próxima vez”.
- Quando apropriado ao objetivo do curso, ajudar o participante a explorar e entender questões comportamentais que podem contribuir com falha de desempenho, como estilo de comunicação,

nothing to be discussed: in a situation of good or excellent performance, the instruction assists the participant in identifying the knowledge, skills and attitudes that contributed to that excellent performance.

Dimension – Element 5
Provide feedback on performance.
When students are learning a complex competence, they sometimes do not have clear understanding their own progress because they are like new learner drivers who may have difficult to master if he/she is on the correct lane. Students need feedback, and this normally means to highlight how they are actually performing and how they are expected to perform. A clear feedback and critical appreciation on how participants’ performance is not consistent, adequate or beyond expectations for a specific simulation activity is crucial for the learning process.

Positive and efficient behaviors include:
- Stating judgments or critiques about participants’’ performance of specific manner – and linked to actions. (Ex. Instead of stating “communication was bad”, you should say “I did not notice that you looked at patient’s eyes or paraphrase what he/she said.”)
- Providing feedback about positive not about negative performance.
- Be direct, but respectful. (Ex. “I didn’t hear you apologize to the patient for his/her mistake. Bear in mind that this may contribute for he/she becomes irritated”; Ex “I didn’t see you double checking the medical prescription. I´m worried because this can increase the risk of medication error.”)

Negative and inefficient behaviors include:
- Talking without reveal opinion or judgment when describing observation on participants’ performance;
- Making biased questions waiting that participants expose performance gap that instructor observed, but did not report.
- Expressing judgment in sarcastic or offensive manner, disrespectful (ex. “I’m not surprised that patient started yelling at you” Why is it so hard to say “I´m sorry”?”)

Explore the source of the performance gaps
Explore rational or movement that forces participants to understand what they are actually doing instead of what they could be is a different characteristic of good debriefing. It is useful to help participants to understand how their knowledge, premises, believes or feelings contribute to their performance. Facilitators should help participants to explore foundations of their actions. Occasionally, there is a need of limit the time or set up a minimal time because the objective of the course can focus only in correct actions instead of help participants to explore and think about their own <u>structures</u>. The debriefing that focus exclusively on actions are normally not classified above 4 and rarely is graded as 5 in this element.

Positive and efficient behaviors include:
- Goes beyond the “what” to the “why”, i.e., explores why participants take actions not focusing exclusively on the do´s and don’ts of each action.
- Encourages participants to reflect on what they were thinking throughout the character playing.
- Provides specific critics linked to actions. Ex. “I didn’t hear you summarizing the mother’s worries”. Or “He/She shrugged, did you notice?”. I believe that, this action contributed with fatigue and discomfort that you felt during laparoscopic procedure. Next time, try to relax your shoulders”.
- When appropriated to the goal of the course, helps the participant to explore and understand behavioral issues that may contribute with performance gap, such communication

planejamento, gestão de carga de trabalho, etc.<br>• Quando apropriado ao curso, ajudar participante a entender as questões sinestésicas ou psicomotoras que contribuíram com falhas de desempenho, como profundidade inadequada de compressões ou técnica inadequada de sutura durante anastomose intestinal.<br>• Quando apropriado ao curso, ajudar participante a entender como seu conhecimento clínico e premissas ou sua aplicação contribuíram para falhas de desempenho, como quais seriam as causas de atraso no diagnóstico e tratamento de embolia aérea.<br>Comportamentos negativos e ineficazes incluem:<br>• Focar somente em corrigir as ações.<br>• Interromper discussão dos processos de pensamento do participante.<br>• Utilizar caracterizações superficiais ou muito abstratas de comportamentos (ex. “Você não ouviu a mãe do paciente”, ex. “Você precisa relaxar mais os ombros assim você não se cansa durante o procedimento.”)<br>• Desdenhar hipóteses dos participantes, conhecimento inadequado ou coordenação física.	style, planning, workload management, etc.<br>• When appropriated to the course, helps the participant to understand kinesthetic or psychomotor issues that contribute to the performance gap, such as inadequate compression depths or inadequate suture technique during intestinal anastomosis.<br>• When appropriated to the course, helps participant to understand how his/her clinical knowledge and premises or its application contribute to performance gaps such as those that would cause late diagnosis and treatment of pulmonary embolism.<br><br>Negative and inefficient behaviors include:<br>• Focusing only on correct actions.<br>• Interrupting discussion on thinking process of the participant.<br>• Using superficial characterization or highly abstract behaviors (ex. You didn’t listen the patient’s mother”, ex. “You need to relax more your shoulder, doing so you’re not become so tired during the procedure.”)<br>• Establishing the hypothesis of participants, inadequate knowledge or physical coordination.
