# Supplementary material for: Translation of the Debriefing Assessment for Simulation in Healthcare in Portuguese and cross-cultural adaptation for Portugal and Brazil
Source: Adv Simul (Lond). 2021 Jul 7;6:25. doi: 10.1186/s41077-021-00175-z (PMC8265112; doi:10.1186/s41077-021-00175-z)
Supplement: Supplementary file 7 — Additional file 7: Supplemental table. Conflicts detected between DASH and Back Translation of Elements 2 and 5. [file 41077_2021_175_MOESM7_ESM.pdf]

Supplemental Table . Conflicts detected between DASH and Back Translation of Elements 2 and 5.

**Conflict detected between Back translation and original document (DASH)**			**Category of the conflict**	**Origin of the conflict**	**Result**
Back Translation	Original (DASH)	Portuguese			
Efficient	Effective	Eficaz	Term	Back translation	No change
Inefficiency	Ineffective	Ineficaz	Term	Back translation	No change
Justifying	Adjusting	Se ajustar	Term	Back translation	No change
Do not notice (or feel)	Don’t appreciate (or resent)	Não apreciam (ou ressentem-se)	Term	Back translation	No change
Limited realism is confused with a problem of solving process.	Limited realism confounds their problem-solving process	Limitação do realismo confunde o processo de resolução do problema	Syntax	Portuguese	Change
Discomfort	Awkwardness	Estranheza	Term	Back translation	No change
To help in the creation of a context of informal conversation	Helps create a context conducive to the conversational probing	Ajuda a criar um contexto de conversa informal	Concept	Portuguese	Change
Communicate on this regard	Communicating such respect	Comunicar esse respeito	Term	Portuguese	Change
Nervous	Hot-headed	Cabeça quente	Term	Back translation	No change
Doing	To make	Fazer	Term	Back translation	No change
Structure	Frame	Estruturas	Concept	Portuguese	Change
Not consistent	Falls short	Está aquém	Term	Back translation	No change

Providing feedback about positive not about negative performance.	Commenting on positive as well as negative performance	Comentar no desempenho positivo e não só no negativo.	Syntax	Portuguese	Change
Did not report	Does not want to say	Não quer dizer	Term	Back translation	No change
Different	Distinctive	Distinta	Term	Portuguese	Change
Movement	Driving force	Força motriz	Term	Portuguese	Change
Throughout the character playing	At the time	Durante o cenário	Concept	Back translation	No change
Correct actions.	Correcting actions	Corrigir as ações	Term	Back translation	No change
Establishing the hypothesis	Sneering at a trainee's assumptions	Desdenhar hipóteses dos participantes	Term	Back translation	No change

Table 2 - (Created from the above table)

19 discrepancies were found distributed as follows:

- 14 (73.6%) related to a Term
- 2 (10.5%) related to Syntax
- 3 (15.8%) related to a Concept

12 (63.2%) discrepancies were seen as originated in the Back Translation and, therefore, did not incur in any necessary changes to the Consensus Harmonized Translation.

- 7 (31.6%) discrepancies were seen as originated in the Forward Translation and that prompted to necessary changes
